# Older adults’ active mobility choices and their specific preferences and needs for their living environment: an intersectional approach

**DOI:** 10.1186/s12889-026-26685-x

**Published:** 2026-03-03

**Authors:** Sophie Horstmann, Sabine Baumgart, Gabriele Bolte

**Affiliations:** 1 University of Bremen, Institute of Public Health and Nursing Research, Department of Social Epidemiology, Bremen, Germany; 2 BPW Stadtplanung, Bremen, Germany

**Keywords:** Intersectionality, Active mobility, Older adults, Urban planning, Heterogeneity, Environment, Walking, Cycling, Healthy aging

## Abstract

**Background:**

For older adults, an effective and accessible way to incorporate exercise into daily life is through active mobility, which includes walking and cycling to everyday destinations. Research highlights the critical role of structural and spatial neighborhood design in promoting active mobility. Nevertheless, most studies on active mobility treat older adults as a homogeneous group, overlooking important differences within this population. An intersectional approach allows researchers to address the specific needs of subgroups that might otherwise go unnoticed. Therefore, this study aims to use an intersectional lens to explore the heterogeneity of active mobility patterns and preferences among older adults in relation to the built environment.

**Methods:**

The cross-sectional study included 2148 older adults (47.0% women) living in eleven rural districts and two urban municipalities (< 100,000 inhabitants) in the Metropolitan Region Northwest in Germany. Data were collected using a self-administered postal questionnaire, pretested with older adults.

Subgroups based on primary mobility mode were identified using conditional inference trees (CIT). We then examined subgroup-specific preferences regarding built environment features influencing mobility choice. Three different sensitivity analyses were performed with complete-case data and two alternative ways to operationalize mobility mode.

**Results:**

We identified eight different subgroups with the most important splitting variables being mobility restrictions, land use mix, and self-reported health status. Across all subgroups, the highest proportion of participants rated the quality of surfaces (59.8% – 84.4%), good lighting (57.3% – 86.4%), and safety concerning traffic (69.7% – 87.3%), and crime (71.4%—90.4%) as important. In general, the subgroup of women with mobility restrictions living in areas with a low land use mix showed the highest proportions of importance ratings.

**Conclusion:**

Our findings highlight the diverse mobility patterns of older adults and their varying environmental preferences and needs. To promote active mobility, urban planning must recognize this heterogeneity and implement tailored, demand-oriented solutions. We demonstrate that a quantitative intersectional approach is a valuable addition to qualitative research for analyzing the combined effects of social identities and living conditions. Future studies should increasingly adopt an intersectional perspective to identify unique experiences of disadvantage and privilege.

**Supplementary Information:**

The online version contains supplementary material available at 10.1186/s12889-026-26685-x.

## Background

The increase in life expectancy and the ongoing global trend of population aging reinforces the need to maintain and promote good health into old age to ensure that individuals can remain mobile, self-determined, and engaged with the world around them as long as possible [1]. Programs for healthy and active aging especially promote physical activity as one of their key aspects [2, 3] since it revealed manifold health benefits for older adults, contributing to both their physical [4, 5] and mental well-being [6]. One effective and accessible way to incorporate exercise into daily life is through active mobility, which is walking and cycling to everyday destinations, such as the grocery store or the doctor’s office [7].

Research has identified the critical role of structural and spatial neighborhood design in promoting active mobility [8], emphasizing the responsibility of urban planning to create environments that support residents’ health and wellbeing [9]. One pressing challenge in urban planning is the development of age-friendly environments that enable active aging, and thus allow older adults to pursue a self-determined life [9, 10]. Given the physical and social changes associated with aging, the mobility patterns and specific needs of older adults often differ from those of younger traffic participants [1]. Hence, to promote active mobility in older adults, a neighborhoods’ design must consider their unique needs and preferences [9].

However, although sharing older age as a common characteristic, it is misleading to treat older adults as one homogeneous group with similar needs [9, 11]. Aging is not a uniform process. Quite the contrary, since everybody ages in various ways, this process is characterized by a high level of diversity [6, 12] leading to a very heterogeneous group of older adults in terms of their health, social positions and experiences [13]. This heterogeneity is also reflected in mobility patterns, choice of transport mode and respectively, the specific requirements and preferences concerning the design of the built environment [1].

Nevertheless, most studies on active mobility treat older adults as a homogeneous group. Some quantitative studies examining active mobility in older adults have considered associations with single aspects such as gender or participant’s mobility restrictions. For instance, a study by van Cauwenberg et al. [8] on physical environmental factors related to walking and cycling in older adults in Belgium indicated that older women have a greater need for safety in traffic compared to older men. Another study by Barnett et al. [14] found positive correlations between pedestrian-friendly neighborhood features, accessible public buildings, and increased walking for transport among Hong Kong older adults with mobility restrictions. Nevertheless, the interconnectedness of multiple aspects has received little attention so far when researching active mobility in older adults.

The theoretical framework of intersectionality promises new insights when researching unequal aging [15]. Coined by Kimberlé Crenshaw in the late 1980 s, intersectionality has gained increasing recognition in health-related research over recent years [16]. The framework provides a valuable perspective for analyzing the combined effects of various social identities — such as gender, socio-economic status, and race — and, thus, allows researchers to identify unique experiences of oppression and privilege and to address the specific needs of subgroups that might otherwise go unnoticed [15, 16].

While qualitative research has begun to incorporate intersectionality into investigations of older adults’ mobility [17–19], the concept remains rarely applied in quantitative studies on aging and mobility. One example is a study by Ang [20], who examined gender and ethnicity in mobility changes in older adults in Singapore. Similarly, Warner and Brown [21] used data from the US Health and Retirement Study to analyze older adults’ age trajectories in functional limitations considering the intersections of gender, ethnicity, and socioeconomic status.

However, both studies relied on predefined intersectional categories and focused primarily on mobility restrictions among older adults rather than on different modes of transport. To our knowledge there is no study using an intersectional lens to conduct an explorative, quantitative investigation of older adults’ mobility. Therefore, this study aims to investigate the heterogeneity of older adults regarding active mobility patterns and preferences concerning the built environment. More precisely we pursued three primary goals: Firstly, we aimed to identify different subgroups of older adults living in small and medium-sized towns and rural communities in Germany based on their mode of transport. Our second goal was to provide a comprehensive description of each of these subgroups to gain an understanding of their specific characteristics. Finally, our third objective was to assess preferences and needs of these subgroups concerning the spatial structure and design of their living environment that influence their choice of transport mode. We hypothesize that these needs and preferences differ significantly between the subgroups.

## Methods

### Study population

The study was conducted as part of the project “AFOOT—Securing Urban Mobility of an Aging Population,” aimed at exploring the mobility behavior of older adults in relation to the built environment. Ethical approval was obtained from the University of Bremen ethical committee (ethics vote 20,181,205).

As part of the project, a cross-sectional postal survey was carried out from May to September 2019. A total of 10,999 adults aged 65 years and older, living in eleven rural districts and two urban municipalities (< 100,000 inhabitants) in the Metropolitan Region Northwest in Germany, were randomly selected by residents’ registration offices. Self-administered paper questionnaires were sent by post with the request to return when filled out. The development of the questionnaire was based largely on validated items from established national and international studies, adapted for use with older adults in Germany. The questionnaire was pretested with 97 older adults from the same area randomly selected by residents’ registration offices. The total response rate was 20.6 percent. Further details on the project, the recruitment strategy, and data collection are published elsewhere [22]. For this analysis, we only included participants living independently and thus, excluded those living in a retirement home (n = 59). Data from 2183 participants was eligible for this analysis. A detailed flow diagram of the selection of the study population is provided in the supplement (supplemental Fig. 1).

### Variables

#### Mode of transport

In line with our first research objective, subgroups within the study population were identified according to their reported mode of transport. The variable was defined as the participant’s primary mode of transport to reach everyday destinations such as the grocery store or the doctor’s office. Participants were asked to indicate on a six-point Likert scale ranging from "daily or almost daily" to "never" how often they typically used various modes of transportation to reach everyday destinations—including walking, cycling, using an electric bike/pedelec, driving a private car, being a passenger in a private car, using a taxi, taking a bus, and riding a train.

Based on this information, we categorized the participants according to their primary mode of transport for the main analysis:

1. Mostly walking: Walking at least three times a week and cycling or using an electric bike/pedelec less often than three times a week.
2. Mostly cycling: Cycling or using an electric bike/pedelec at least three times a week and walking less often than three times a week.
3. Walking and cycling: Walking and cycling both at least three times a week.
4. Car-centric: Using a private car as a driver or passenger at least three times a week, and both, walking and cycling, less often than three times a week.
5. Not very active: Every mobility option less than three times a week.
6. Not active at all: Every mobility option less than one to two times a week.

The frequency of car or public transport use did not influence the categorization into groups one to three.

To be included in the analyses, participants had to provide information on their usual use of at least one mode of transport. In total, 31 participants were excluded due to missing data. In this rural and small-town study population, only four participants primarily used public transport to reach everyday destinations. As the number of cases was too small for further analyses, these participants were excluded resulting in a study population of 2,148 participants.

For the sensitivity analyses, we constructed two alternative variables to describe participants' mode of transport. First, we created a binary variable distinguishing between participants who engage in active transportation (walking and/or cycling) at least three times a week and those who do so less frequently.

Second, we defined a four-category variable. Categorization was performed without considering the frequency of using the car or public transport:

1. Mostly walking: Walking at least three times a week and cycling or using an electric bike/pedelec less often than three times a week.
2. Mostly cycling: Cycling or using an electric bike/pedelec at least three times a week and walking less often than three times a week.
3. Walking and cycling: Walking and cycling both at least three times a week.
4. No active mobility: Walking or cycling less often than three times a week.

In order to identify the subgroups and further describe them in accordance with our second research objective, we collected information on participants’ sociodemographic characteristics and living situation, access to a car, health, and neighborhood environment.

#### Sociodemographic characteristics and living situation

Participant’s gender was recorded by a dichotomous variable, distinguishing between 'man' and 'woman'. We categorized participants into four different age groups (‘65–69 years’, ‘70–74 years’, ‘75–79 years’, ‘80 years and over’) based on their reported year of birth. Participants' highest school qualification and professional education was categorized into three different levels according to the International Standard Classification of Education (ISCED) [23]: ‘low’ (ISCED 0–2), ‘middle’ (ISCED 3–4), and ‘high’ (ISCED 5–6). The equivalized income indicates where the participant’s equivalized disposable household income falls in relation to the median income in Lower Saxony at the time of the study [24]: " < 60% of the median", " ≥ 60% of the median and ≤ median", and " > median”. Based on information on household composition, we distinguished participants’ living situation into ‘living alone’ (one household member) or ‘not living alone’ (more than one household member).

#### Access to car and driver’s license

For each participant we assessed if they have a driver's license and access to a car. To determine car access, we aggregated participants who stated they had "at any time" or "occasionally" access to a car as a driver or passenger.

#### Health, mobility restrictions, and use of walking aids

We categorized participants based on their self-rated health into two groups: individuals with a 'very good' or 'good' health, and those with a 'moderate,' 'poor,' or 'very poor' health. Participants were queried about potential restrictions in mobility due to health issues, comprising difficulties in walking, seeing, or other impairments. Those reporting at least one of these issues were classified as having mobility restrictions. Use of walking aids was assessed by asking the participants if they used any walking aids with the possibility to further specify as walking stick, crutches, walker, walking frame, quad walker and/or a wheelchair.

#### Neighborhood environment

Questions regarding participants' neighborhood environment were derived from the Neighborhood Environment Walkability Scale [25, 26]. While most questions remained unchanged from the original instrument, some were adapted to better suit the German context. For precise details on the specific items per subscale and their wording, please refer to supplemental Table 1.

The land use mix represents the diversity of everyday destinations accessible within walking distance from a participant's home. To calculate a land use mix score we took the mean value of participants’ estimates of the time it takes them to walk to various destinations, such as grocery stores, physicians, recreation centers, using a four-point scale: 1–10 min, 11–20 min, 21–30 min, and more than 30 min.

We calculated subscale scores to assess participants' ratings of their neighborhood environment. Using a 4-point scale ranging from 'strongly agree' to 'strongly disagree,' participants indicated their agreement with statements that reflected various aspects of their neighborhood, including:

- Walking and cycling infrastructure: How well the neighborhood’s spatial and structural design supports walking and cycling, respectively.
- Street connectivity: How well streets, roads, and pathways are integrated within the transportation network, providing direct and efficient routes for commuting.
- Aesthetics: How attractive the neighborhood is.
- Traffic safety: How safe the streets are in terms of traffic-related hazards.
- Crime safety: How safe the neighborhood is in terms of crime.

Subscale scores were calculated by taking the mean of the relevant items. Crime safety in the neighborhood was assessed using a single item.

Area of residence indicates the population size of participants’ urban district or municipality. We defined four categories according to the classification provided by the Federal Institute for Research on Building, Urban Affairs, and Spatial Development: ‘medium-sized town’ (20,000–99,999 inhabitants), ‘larger small town’ (10,000–19,999 inhabitants), ‘small town’ (5,000–9,999 inhabitants or possessing at least basic central functions), and ‘rural community’ (fewer than 5,000 inhabitants) [27]. A characterization of the four categories of municipalities (according to their population size) concerning the degree of land use mix is given in supplemental Fig. 2.

In line with our third objective, we assessed participants’ preferences and needs regarding the spatial structure and design of their living environment. To measure participants’ preferences for the design of their spatial and structural neighborhood environment they were asked to rate the importance of 21 environmental features using a 4-point Likert scale: 'important', 'fairly important', 'fairly unimportant', and 'unimportant'. Moreover, participants were presented with 18 potential reasons for their choice of mode of transport, which they rated on a 4-point Likert scale: 'important', 'fairly important', 'fairly unimportant', and 'unimportant' to indicate how much the specific reason applied to them.

### Statistical analyses

Descriptive analyses were performed for the total study population and stratified by gender. We calculated absolute and relative frequencies for age, education, equivalized income, self-reported health, mobility restrictions, and area of residence. We plotted histograms for land use mix stratified by area of residence.

#### Identification of different subgroups based on participants’ mode of transport

To identify subgroups, we used the recursive partitioning algorithm CIT, initially developed by Hothorn et al. [28]. This statistical method grows a decision tree that divides the population into subgroups based on dichotomized independent variables. The algorithm first selects the covariate with the strongest bivariate association with the dependent variable. It then determines the optimal threshold of the chosen covariate to split the population into two groups.

The dependent variable for our analysis was mode of transport. We chose potential splitting variables based on previously published evidence: gender, age, education, equivalized income, living situation, self-rated health, mobility restrictions, land use mix, walking infrastructure, cycling infrastructure, street connectivity, aesthetics, traffic safety, crime safety and area of residence. For details on all splitting variables please see supplemental Table 2. We set the threshold for variable selection to 0.05 and used a Bonferroni correction to adjust for multiple testing. The algorithm stops if none of the covariates shows a significant association with the dependent variable, or if the stopping criteria of a minimum node size of 50 and a maximum depth of 20 are met.

The CIT algorithm does not require a complete case analysis. When identifying the best splitting variable, observations with missing values in the covariate under investigation are ignored. To assign observations with missing values to the best fitting child nodes, surrogate splits are identified to mimic the decision rule as closely as possible [28]. We set the number of surrogate splits to evaluate at a maximum of three. To gain deeper insights into the identified subgroups, we used the algorithm to identify the variables used for surrogate splits (supplemental Fig. 3).

To improve the robustness of the results and reduce the risk of overfitting, we used a random forest approach. In this method, decision trees are grown using a randomly selected subsample of the original data and a random subset of variables. The results of all trees are then combined, allowing to identify the variables that are most important across the forest and to compare them with the splitting variables identified in the original CIT [24]. In our analysis, we grew a random forest consisting of 1,000 trees [29].

#### Description of each subgroup and environmental preferences and needs

For every subgroup, we calculated frequencies of mobility type, age, education, equivalized income, gender, drivers’ license, access to a car, and living situation. Additionally, we calculated frequencies of the different types of mobility restrictions and plotted the frequency of car use for individuals with mobility restrictions, frequencies of participants’ use of public transport services, and use of walking aids.

We visualized participant’s preferences for the design of the spatial and structural neighborhood environment and reasons for choice of mode of transport using radar charts, stratified by subgroup.

### Sensitivity analysis

To test if the most important splitting variables, the identified subgroups and their preferences and needs remain consistent across different analyses, we repeated our approach for subgroup identification and visualization of neighborhood environment preferences and reasons for mode of transport choice across three sensitivity analyses: First, we conducted a complete-case analysis to evaluate the impact of missing data on our results. This analysis included only the 1,618 participants with no missing values for any potential splitting variable or the dependent variable. Additionally, we examined how different categorizations of the dependent variable effect the findings by using a mobility variable with either two or four categories as described above.

All statistical analyses were conducted in consultation with a statistician. Analyses and diagrams were compiled with R version 4.3.2.

## Results

In total we included 2148 participants into the analysis of whom 47.0% were women and 53.0% men. Mean age of the population was 74.5 years. Sociodemographic and health-related characteristics of the study population are presented stratified by gender in Table 1 (supplement Table 2).

**Table 1:** Sociodemographic and health-related characteristics of the study population

	**Total**	**Gender**	
		Women	Men
	N (%)	N (%)	N (%)
	2148 (100)	976 (47.0)	1099 (53.0)
Age, years			
65–69	584 (28.2)	285 (29.2)	299 (27.3)
70–74	531 (25.6)	257 (26.3)	273 (24.9)
75–79	497 (24.0)	230 (23.6)	267 (24.3)
80 +	462 (22.3)	204 (20.9)	258 (23.5)
*missings*	*74*		
Education, ISCED			
low	165 (7.8)	126 (13.1)	34 (3.1)
middle	1207 (57.0)	622 (64.5)	536 (49.4)
high	750 (35.3)	217 (22.5)	514 (47.4)
*missings*	*26*		
Equivalized income			
< 60% median	280 (13.4)	130 (13.8)	143 (13.4)
60%—median	688 (33.0)	328 (34.8)	332 (31.0)
> median	1118 (53.6)	485 (51.4)	596 (55.6)
* missings*	*62*		
Self-rated health			
very good/good	1244 (58.5)	560 (58.0)	631 (58.1)
moderate/poor/very poor	881 (41.5)	406 (42.0)	455 (41.9)
* missings*	*23*		
Mobility restrictions			
yes	812 (38.3)	385 (40.2)	411 (37.7)
no	1307 (61.7)	572 (59.8)	680 (62.3)
*missings*	*29*		
Area of residence			
medium-sized town	644 (30.4)	312 (32.4)	316 (29.1)
larger small town	779 (36.7)	360 (37.3)	385 (35.5)
small town	538 (25.4)	224 (23.2)	297 (27.4)
rural community	160 (7.5)	68 (7.1)	87 (8.0)
*missings*	*27*		

Table 2 presents the distribution of the six categories of mode of transport stratified by gender. The tree modes of transport—mostly walking, walking and cycling, and car-centric – are equally represented in the study population, each accounting for approximately 25%. A total of 14.9% of participants mostly cycle for transportation, while the least active groups—those who are not very active (6.8%) or not active at all (2.1%)—make up the smallest percentages. A similar distribution can also be observed when looking on women and men separately.

**Table 2:** Frequencies of mode of transport stratified by gender

	Total (N = 2148)	Gender	
		Women (*N* = 976)	Men (*N* = 1099)
	N (%)	N (%)	N (%)
mostly walking	552 (25.7)	256 (26.2)	280 (25.5)
mostly cycling	319 (14.9)	151 (15.5)	157 (14.3)
walking and cycling	570 (26.5)	219 (22.4)	321 (29.2)
car-centric	516 (24.0)	237 (24.3)	267 (24.3)
not very active	147 (6.8)	91 (9.3)	53 (4.8)
not active at all	44 (2.1)	22 (2.3)	21 (1.9)

### Identification of subgroups based on participants’ mode of transport

**Fig. 1:**
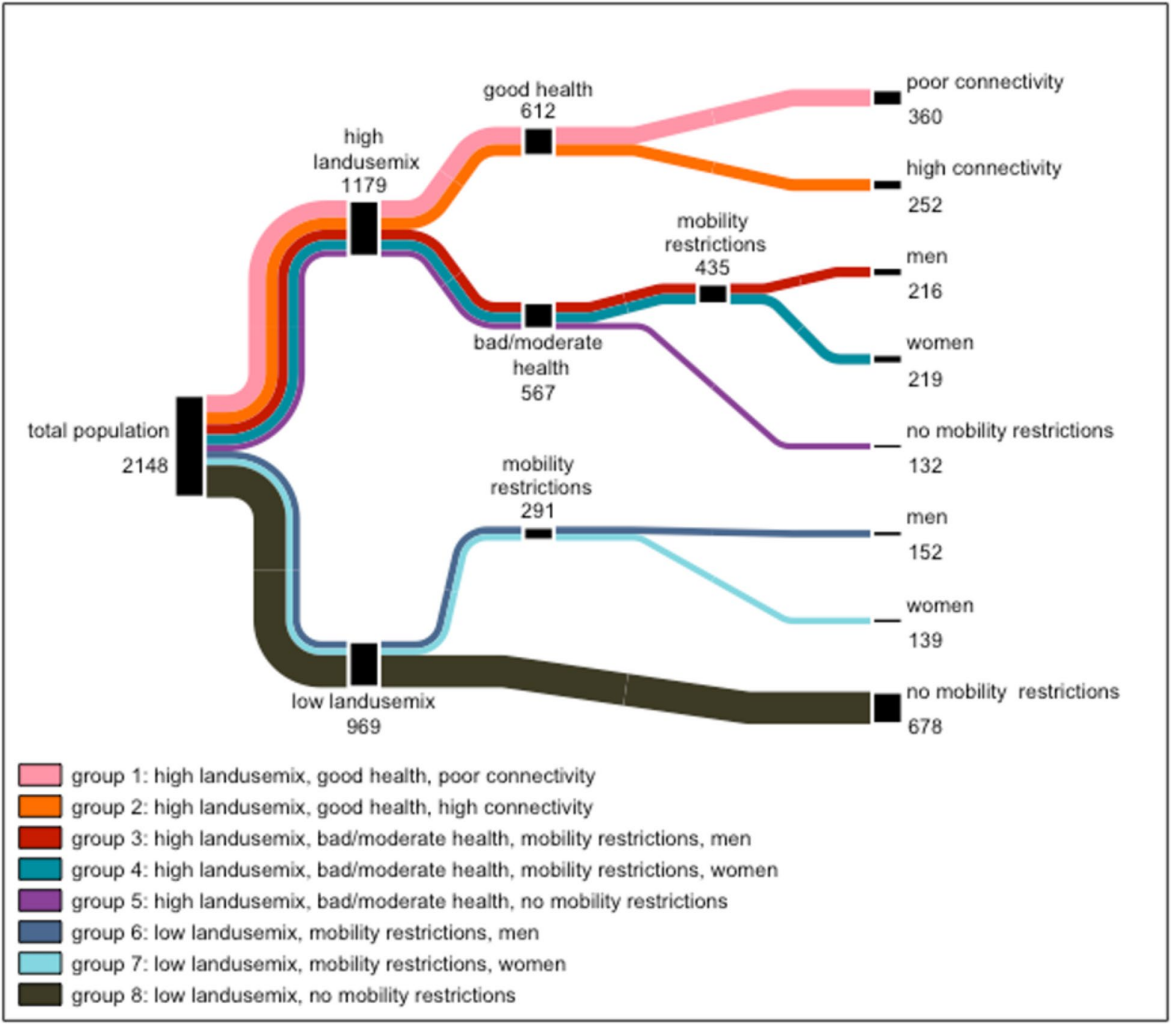
Subgroup distribution based on participant’s primary mobility mode. High land use mix: ≤ 2.632, low land use mix > 2.632; Poor connectivity ≤ 2.333, high connectivity > 2.333 thresholds are identified by the algorithm as optimal split of the population

Running a CIT analysis for participant’s mobility mode resulted in a decision tree with 15 splits and eight different subgroups (Fig. 1).

The first split was initiated by the land use mix in participants' neighborhoods, using a threshold of 2.632. Participants living in areas with a high land use mix were sent to one branch of the tree to be split again by their self-rated health status. Participants who reported to have a good or very good health were further divided based on the connectivity level of their neighborhood environment with a threshold of 2.333. This resulted in Group 1, formed out of 360 participants who reported to have a good health status, situated in a neighborhood environment with a high land use mix but poor street connectivity. Group 2 consists of 252 participants with a good health status living in a neighborhood environment characterized by both a high land use mix and high street connectivity.

Participants who rated their health as poor or moderate were sent to one branch if they reported having mobility restrictions and were then further divided based on their gender. Thus, three different subgroups were formed: Group 3 and Group 4, both comprising participants living in neighborhoods characterized by a high land use mix, reporting poor or moderate health, and indicating mobility restrictions. Group 3 exclusively includes men (n = 216), while Group 4 solely contains women (n = 219). Group 5 comprises 132 participants living in areas with a high land use mix who reported having poor or moderate health but no mobility restrictions.

Following the first split, participants living in a neighborhood environment characterized by a low land use mix (≤ 2.632) were sent to another branch. Here, they were split again based on mobility restrictions and gender. This led to three further subgroups: Group 6 and Group 7 comprised men (n = 152) and women (n = 139), respectively, who live in neighborhoods with low land use mix and reported having mobility restrictions. Group 8—the largest subgroup with a total of 678 participants—consisted of individuals living in areas with a low land use mix who have no mobility restrictions.

**Fig. 2:**
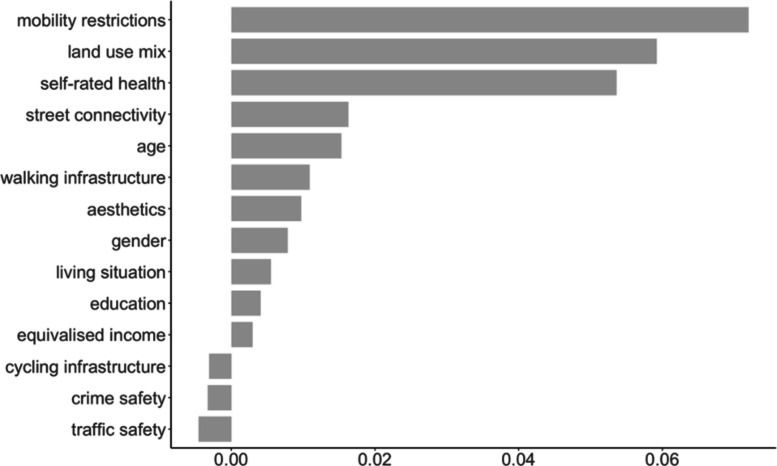
Variable important measures

Conducting a test for variable importance by growing a random forest with 1000 trees identified mobility restrictions, land use mix, and self-reported health status as the three most important variables (Fig. 2).

### Description of each subgroup

**Fig. 3:**
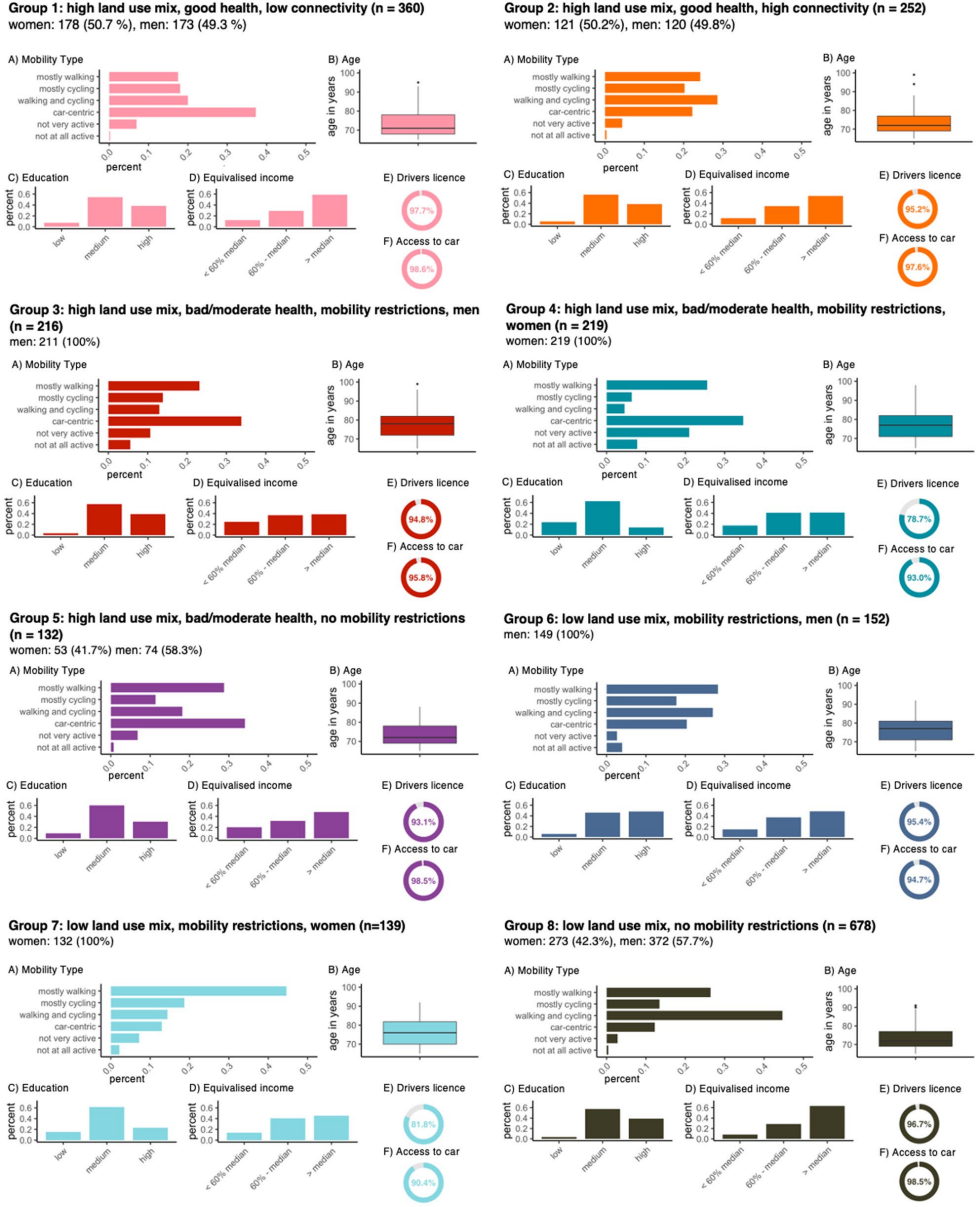
Descriptive analysis of the eight subgroups. To calculate the proportions for the different variables, we excluded missing values

Figure 3 and supplemental Table 2 show the distribution of main characteristics of the eight subgroups. Supplemental Table 3 shows the type and number of mobility restrictions per group while supplemental Fig. 4 illustrates the frequency of car use in individuals with mobility restrictions. Supplemental Figs. 5 and 6 visualize the subgroups’ use of public transport services and walking aids, respectively.

Group 1 consists of women (50.7%) and men (49.3%) who are in good health and live in areas with a high land use mix but poor connectivity. The most used main mode of transport is the car (37.2% of participants). Compared with the other groups only a small percentage of participants primarily walks (17.5%). The majority of participants in this group has a medium level of education according to the ISCED classification (54.2%), and 58.8% of participants have an equivalized income higher than the median household income in Lower Saxony at the time of the study.

Group 2 consists of 50.2% women and 49.8% men, with participants in good health living in areas with a high land use mix and high connectivity. Compared to other groups, Group 2 comprises a relatively high number of participants who mostly cycle (20.2%), or walk and cycle (28.6%) to reach everyday destinations. Additionally, a relatively high number of participants in this group have an equivalized income above the median (53.7%).

In Group 3, which comprises men living in neighborhoods with high land use mix, reporting poor or moderate health, and indicating mobility restrictions, the most used mode of transportation is the car (33.8%). 63.4% of participants in Group 3 stated having walking impairments, and among these individuals, almost 40% drive a car at least three times a week. In this group, the number of people with an equivalized income between the poverty line (60% of the median) and the median is nearly equal to those with an income above the median, with relative frequencies of 36.8% and 38.7%, respectively.

Group 4, consisting of women with poor or moderate health and mobility restrictions living in areas with a high land use mix, includes the oldest participants on average, with a mean age of 77.4 years. This group also shows the highest percentages of participants who are not very active (20.9%) or not active at all (7.7%). The highest percentage of participants in this subgroup (34.5%) uses a car as their main mode of transport as driver or passenger. Nearly a quarter (23.7%) of participants in this group have a low level of education, the highest percentage in this category compared to the other groups. Additionally, the number of people with an equivalized income between the poverty line (60% of the median) and the median is equal to those with an income above the median (41.3%). Compared to the other groups of this study population, Group 4 comprises a relatively moderate percentage of participants with a driver's license (78.8%) and the highest percentage of participants living alone (33.8%).

Group 5 consists of 41.7% women and 58.3% men living in areas with a high land use mix who reported having poor or moderate health but no mobility restrictions. Among the participants, 34.1% take the car to reach everyday destinations, while 28.8% walk. The majority of participants in this group have a medium level of education (60.3%).

Comprising men with mobility restrictions living in areas with a low land use mix, participants in Group 6 primarily walk (28.3%) or walk and cycle (27.0%) to reach everyday destinations. Unlike the other groups, in Group 6 the proportion of participants with a high level of education is rather high (48.0%). Those with a medium level of education account for 46.0%.

Group 7 consists of women with poor or moderate health and mobility restrictions living in areas with a high land use mix. The highest percentage of participants in this group walks to reach everyday destinations (44.2%). Similar to Group 4, Group 7 comprises a relatively small percentage of participants with a driver's license (81.6%). Additionally, 62.0% of the participants in Group 7 have a medium level of education according to the ISCED classification, while the number of participants with a high level of education is relatively small compared to the other groups (22.6%).

In Group 8, comprising individuals without mobility restrictions living in neighborhoods with a low land use mix, participants are the youngest, with a mean age of 72.9 years. Almost half of the participants (44.7%) in this group reported walking or cycling at least three times a week for active mobility. The group consists of 42.3% women and 57.7% men, with a high number of participants having a disposable income higher than the median (63.0%) compared to the other groups.

### Preferences and needs concerning the design of the neighborhood environment

**Fig. 4:**
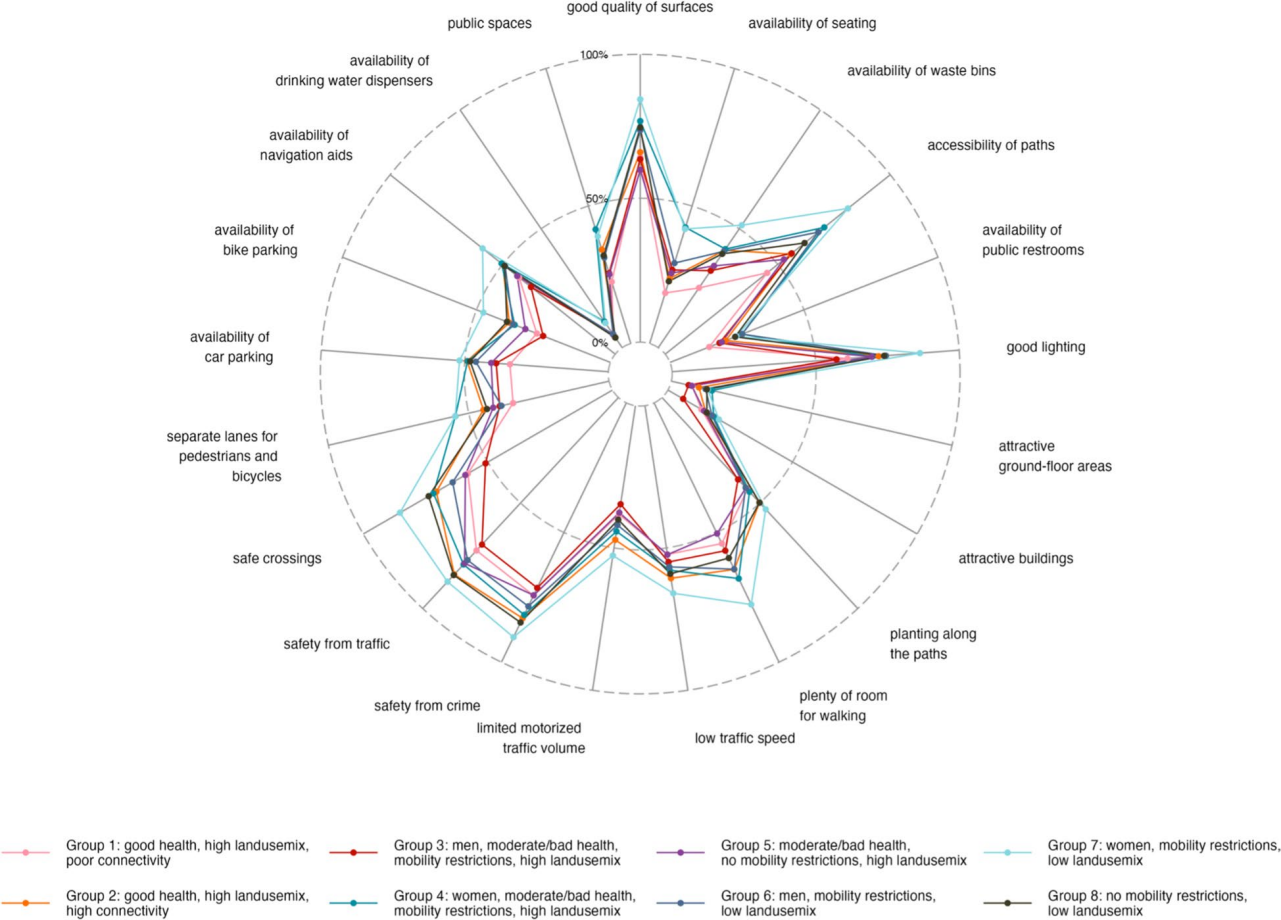
Proportion (%) of participants per subgroup who rated features of their structural and spatial neighborhood environment as important

Figure 4 and supplemental Table 5 illustrate the proportion of participants per subgroup who rated features of their structural and spatial neighborhood environment as important. Across all subgroups, the highest proportion of participants rated the quality of surfaces (59.8% – 84.4%), good lighting (57.3% – 86.4%), and safety concerning traffic (69.7% – 87.3%) and crime (71.4%—90.4%) as important. In contrast, the lowest proportion perceived the attractiveness of the neighborhood environment (6.1% – 14.5%) and the provision of water dispensers (4.2% – 11%) as important. Overall, women with mobility restrictions who live in areas with a low land use mix (Group 7) showed the highest proportions, particularly emphasizing the importance of good walking infrastructure and safety regarding traffic, crossings, and crime. The lowest proportions of importance ratings were observed among participants with a good health living in areas with a high land use mix but poor connectivity (Group 1) and men with a bad or moderate health and mobility restrictions who live in an area with a high land use mix (Group 3).

**Fig. 5:**
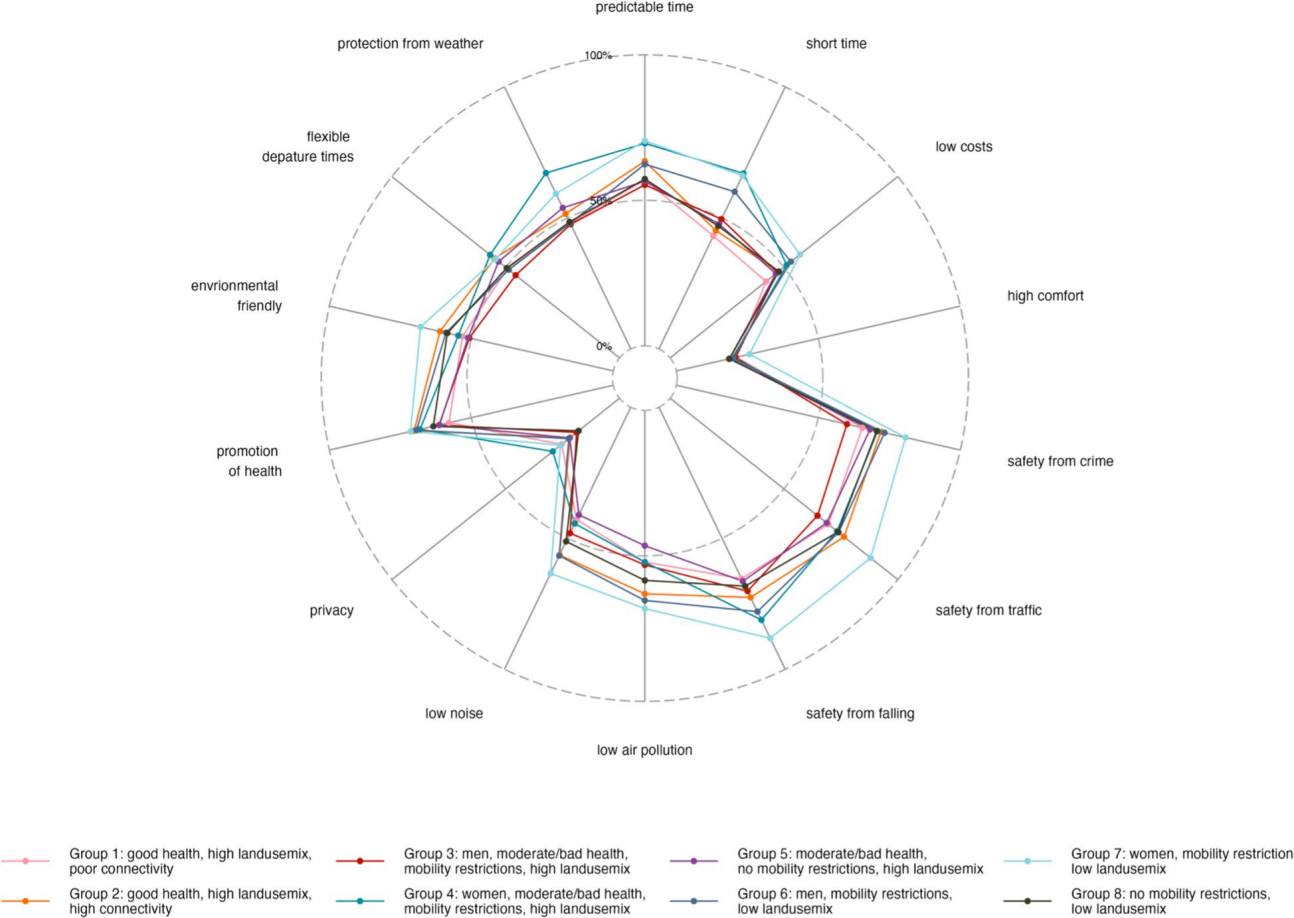
Proportion (%) of participants per subgroup who rated various features of the neighborhood environment as important when choosing their mode of transport

Figure 5 and supplemental Table 6 show the percentage of participants per subgroup who rated various features of the neighborhood environment as important when choosing their mode of transport. Women with mobility restrictions living in areas with a low land use mix (Group 7) showed the highest proportions. They particularly highlighted safety aspects regarding crime (80.7%), traffic (88.1%), and the risk of falling (88.1%) as important when selecting their mode of transport. Both groups restricted to women, Group 4 and Group 7, prioritized predictable (69.6% and 70.5%, respectively) and short journey times (67.0% and 66.2%, respectively). Participants across all groups reported that they consider positive effects on their health (57.9% – 71.4%) and the environment (50.7% – 67.9%) when choosing their mode of transport, although to varying degrees. Privacy (18% – 29.3%) and comfort (18.5% – 25.8%) were the least important features for all groups.

### Sensitivity analyses

Similar to the main analysis, all three decision trees from the sensitivity analyses initially split based on the land use mix of the participant’s neighborhood. However, unlike the main analysis, decision tree models using complete cases and a four-category classification of transportation modes resulted in fewer splits, initiated by land use mix and education level (supplemental Figs. 7–9), and land use mix and connectivity (supplemental Figs. 10–12), respectively. A binary classification of transportation mode produced a pattern closely resembling the main model, with eight distinct subgroups (supplemental Figs. 13–15).

Across the three sensitivity analyses, land use mix and street connectivity consistently showed high variable importance measures when growing random forests.

Regarding participant’s preferences and needs concerning the design of the neighborhood environment the patterns of the three sensitivity analysis resembled the ones identified in the main model. Overall, safety aspects and the quality of the spatial and structural neighborhood environment were rated as important by most participants. Please refer to the supplement for the decision trees and radar charts.

## Discussion

In this study, we conducted an explorative analysis using an intersectional lens to capture the heterogeneity of older adults regarding active mobility patterns and preferences concerning the built environment. We used decision tree analysis to identify different subgroups of older adults based on their primary mode of mobility. Participant’s health and mobility restrictions, and aspects of the spatial and structural neighborhood design such as land use mix and street connectivity were of high importance as splitting variables. Across all subgroups, the highest proportion of participants rated aspects concerning safety from traffic and crime as important when choosing their mode of transport.

By applying decision tree analysis to conduct an explorative investigation of older adults’ heterogeneity in active mobility patterns, this study demonstrates the applicability of quantitative methods as a valuable addition to the existing evidence base provided by qualitative intersectionality-informed research [17–19, 30]. To our knowledge, our study is among the first quantitative investigations to apply an intersectional framework to examine older adults’ mobility patterns and their needs and preferences regarding the design of the spatial and structural environment in greater depth.

Urban design criteria must match with residents’ characteristics and abilities [1, 9, 31, 32]. However, systems of power determine whose data is collected and analyzed and who is visible and prioritized in urban planning decisions [33]. As a result, the design of the environment itself can become a source of inequality [34] empowering some groups while marginalizing others [32, 35]. Urban planning has over the past decades been strongly influenced by the perspectives and needs of the working population, resulting in structures that are often organized around motorized commuting to and from work [36, 37]. This focus can disadvantage older adults, whose mobility patterns and needs often differ from those of younger traffic participants due to age-related physical and social changes [1]. The Age-Friendly Cities framework—initiated by the World Health Organization—seeks to integrate older adults’ diverse perspectives, needs, and capacities into urban planning processes to promote accessible and inclusive structural and spatial design [1, 9, 31, 32]. To achieve this, scholars increasingly emphasize the importance of reliable data and evidence to inform decision-making, since especially the perspective of specific subgroups in older adults still remains underrepresented when making urban planning decisions [33, 38]. In the long term, the use of intersectionality-informed quantitative approaches to examine heterogeneity among older adults as used in our study may help increase the visibility of distinct subgroups and their specific preferences and needs in urban planning decisions.

In their guide on integrating an intersectional approach into urban planning, Williams et al. [34] emphasize the importance of recognizing how systems of power shape individuals’ experiences, potentially conferring benefits on some groups while disadvantaging others. Thus, future studies should build on our research with complementing analyses of how the different subgroups of older adults are placed within interlocking systems of oppression—such as racism, sexism, and classism [16, 39, 40], recognizing age itself is an axis of inequality [38, 41].

Since privilege and disadvantage depend on time and space [42] the subgroups and their specific preferences we have identified might differ from those in other contexts. What is more, we focused with our analysis on the direct neighborhood environment. Moving further away from home—such as in the whole community, or the surrounding area—requires different skills [31, 43]. Thus, in the future research should put a special focus on analyzing older adults’ needs and preferences concerning the spatial and structural design of their environment in different contexts and mobility settings [43].

Since rural communities pose different challenges then cities when creating age-friendly environments [44], scholars argue that the age-friendly cities framework’s focus on urban contexts risks overlooking the diverse and potentially distinct needs of rural communities. We examined mobility and neighborhood preferences among older adults living independently in small- and medium-sized towns and rural communities in Germany to address the gap in systematically assessing and incorporating the perspectives of older adults living in rural areas when creating age-friendly environments [44, 45]. As one of our main findings we identified the importance of land-use mix and connectivity in shaping different mobility subgroups among older adults living in rural areas. Our results align with findings from a previous systematic review from Cerin et al. [46] reporting strong associations between land-use mix, street connectivity, and walkability, and older adults’ participation in active mobility, including walking and cycling.

In general, we found a high percentage of car use as driver or passenger. Oxley and Whelan [1] state that many older adults have more problems with other modes of mobility than they have with driving. This statement is consistent with our own findings showing that a high percentage of participants with mobility restrictions drove a car regularly within one week.

We observed, however, that women with mobility restrictions living in neighborhoods with a low land use mix mostly walked to reach their everyday destinations. Since this group showed the lowest percentage of participants with a driver’s license and the highest proportion of those living alone, many women in this subgroup may have no alternative but to walk to their everyday destinations. The specific group of older adults without a driver’s license and with limited or no access to travel as passengers has received little attention in the existing literature. Though, research on cessation of driving in older adults in general showed an association with a higher risk of social isolation among those giving up driving [47–49]. Particularly in rural contexts with low land use mix and limited street connectivity, distances may be too far for older adults’ walking or cycling capacities, highlighting the need for alternative modes of transport to support continued participation in everyday life, even when they do not have access to a car or decide to cease driving.

As an alternative for driving, research on age-friendly urban planning emphasize the importance of public transport [9, 44] and the potential of smart mobility solutions for age-friendly environments [38]. Our study was performed in an area, where only limited public transport services are available. As a result, most participants did not regularly use public transport services and we could not add a category of predominantly public transport use to our mobility variable. Further investigations should also adopt an intersectional perspective to examine the capacities and needs of different subgroups of older adults regarding alternative modes of transport, and how to design solutions that are accessible and usable for older adults with diverse abilities in rural contexts [44, 48].

Another of our findings is that men with mobility restrictions engaged in a wider range of active mobility activities, including both walking and cycling, whereas women with mobility restrictions primarily walked. One possible explanation for this gender difference may lie in variations in safety perceptions. In line with this interpretation, a cross-sectional study by van Cauvenberg et al. [8] examining the relationship between physical environmental factors and walking and cycling among older adults in Belgium found that feelings of unsafety were associated with a decreased likelihood of daily cycling for transportation among women, but not among men. However, these findings were not stratified by mobility restrictions. Extending this evidence, our data shows that although safety from crime and traffic was considered important across all participants, it was particularly emphasized by women with mobility restrictions living in neighbourhoods with low land use mix when choosing their mode of mobility.

Another possible explanation for the observed gender differences in active mobility among individuals with mobility restrictions may lie in gendered variations in confidence regarding physical capacity. Walking requires less confidence than other forms of physical activity such as cycling [50]. Additionally, research has shown that participants who viewed themselves as more autonomous tended to navigate larger areas with older men reporting a greater sense of autonomy than women [51]. Similarly, low self-efficacy has been linked to more restricted activity spaces [43]. Although our questionnaire did not assess self-efficacy directly, our findings align with current literature. Women with mobility restrictions rated the availability of seating as more important than other groups and placed greater emphasis on predictable and short travel times. Moreover, safety from falling was considered important across all groups, particularly among women with mobility restrictions.

Given that ageing is a gradual process rather than a discrete event [39] current discussions in age-friendly environments research emphasize the value of adopting a life-course perspective to better capture individual experiences of ageing [30, 52]. Building on this perspective and our findings, future research should examine how ageing as a member of a specific gender group and within a gendered environment shapes older adults’ mobility, as well as their perceptions of safety and self-assessed capacities. Furthermore, future research should investigate how existing spatial structures influence perceptions of safety and physical capabilities among different subgroups of older adults when participating in traffic. Adding to current discussions on age-friendly environments [53] our analysis highlights the importance of street connectivity, pedestrian crossings designed to accommodate older adults (e.g., in terms of crossing time and visibility), and the availability of well-maintained walking and cycling paths. Since our study focused on the aspects of the living environment that participants considered important when participating in traffic, we focused our research on older adult’s subjective perceptions of their neighborhood environment. However, previous research has shown that perceived and objective environments might differ [54–56]. Incorporating objective measures in future work would provide a more comprehensive understanding — particularly when examining structural inequalities and their intersections with spatial design.

### Limitations and strengths

There are some limitations of our study that have to be considered when interpreting our findings. Data was collected using a cross-sectional study design, which does not allow for determining the chronological sequence of events. As a result, it remains unclear whether participants’ preferences regarding their structural and spatial environment influenced their mobility decisions or if their mobility habits shaped their preferences and informed their desires for their surroundings.

Data were collected using a self-administered questionnaire that was mailed to randomly selected residents, who were asked to return the completed questionnaire by mail. Consequently, there is a potential risk of selection bias. When compared with the general population of Lower Saxony at the time of data collection, the distributions of education level, household income, country of birth, and residential area were comparable to those in our study population. In contrast, our sample included a higher proportion of men, married individuals, and respondents reporting good or very good health. Therefore, the findings should be interpreted with caution regarding their generalizability [22].

The questionnaire was primarily based on validated items, with some adaptations to suit our study population of older adults in Germany. We pretested the questionnaire before use with focus groups of older adults [22]. However, the validity of certain items for this specific population remains uncertain. In addition, data were collected through self-report, requiring participants to estimate their daily travel times and distances to everyday destinations. Therefore, the findings should be interpreted carefully due to the potential of information bias. With our study we focused on the association between aspects of the built environment and older adults’ mobility. Thus, no information on how participants perceive their own physical capabilities were gathered.

Moreover, participant’s gender was assessed via a binary variable distinguishing between men and women. Although this method of recording does not allow for a precise distinction between the different dimensions of sex and gender [57] we could identify significant differences in our study population that might be connected with the social dimensions of gender – such as differences in the frequency of holding a driver’s license and the perceived importance of safety aspects. Thus, within our publication, we decided on using the term”gender” to refer to gender aspects entangled with the biological dimension of sex. However, the findings on the binary categories—men and women—must be interpreted with caution and more thorough studies using various dimensions of sex and gender are warranted.

This study also has a number of strengths that set it apart from others. It is one of the first quantitative studies to examine active mobility in older adults through an intersectional lens. By conducting an explorative analysis, we aimed to identify hitherto unknown patterns and relationships, shedding light on a largely overlooked research area. The use of decision tree analysis enabled the simultaneous consideration of multiple variables, helping to uncover hidden connections within the data. In addition, most studies on active mobility in older adults are conducted in urban environments. This is one of the first studies focusing on rural districts and small- to medium-sized towns with < 100,000 inhabitants and thus generating information on active mobility in older adults living independently in less densely populated areas.

## Conclusion

Our findings emphasize the diverse mobility patterns of older adults and their varying environmental preferences and needs. To promote active mobility, urban planning must recognize this heterogeneity, examine, critically, older people’s travel needs and abilities, and implement tailored, demand-oriented solutions. We demonstrated that a quantitative intersectional approach is a valuable addition to qualitative research for analyzing the combined effects of social identities and living conditions. Future studies should increasingly adopt an intersectional perspective to identify distinct experiences of disadvantage and privilege. In doing so, they should place particular emphasis on combining individual- and structural-level factors in their analyses to identify entry points for promoting active mobility for all subgroups of older adults.

## Supplementary Information

Supplementary Material 1.

## Data Availability

The datasets analyzed during the current study are not publicly available, as the informed consent signed by participants specifies that the data will not be shared with third parties. However, the questionnaire (in German language) and aggregated data are available from the corresponding author on reasonable request.
