# Supplementary material for: Older adults’ active mobility choices and their specific preferences and needs for their living environment: an intersectional approach
Source: BMC Public Health. 2026 Mar 3;26:1145. doi: 10.1186/s12889-026-26685-x (PMC13063558; doi:10.1186/s12889-026-26685-x)
Supplement: Supplementary file 1 — Supplementary Material 1. [file 12889_2026_26685_MOESM1_ESM.pdf]

Older adults' active mobility choices and their specific preferences and needs for their living environment: an intersectional approach

Supplement

Sophie Horstmann[1], Sabine Baumgart[1,2], Gabriele Bolte[1]

1 University of Bremen, Institute of Public Health and Nursing Research, Department of Social Epidemiology, Bremen, Germany
2 BPW Stadtplanung, Bremen, Germany

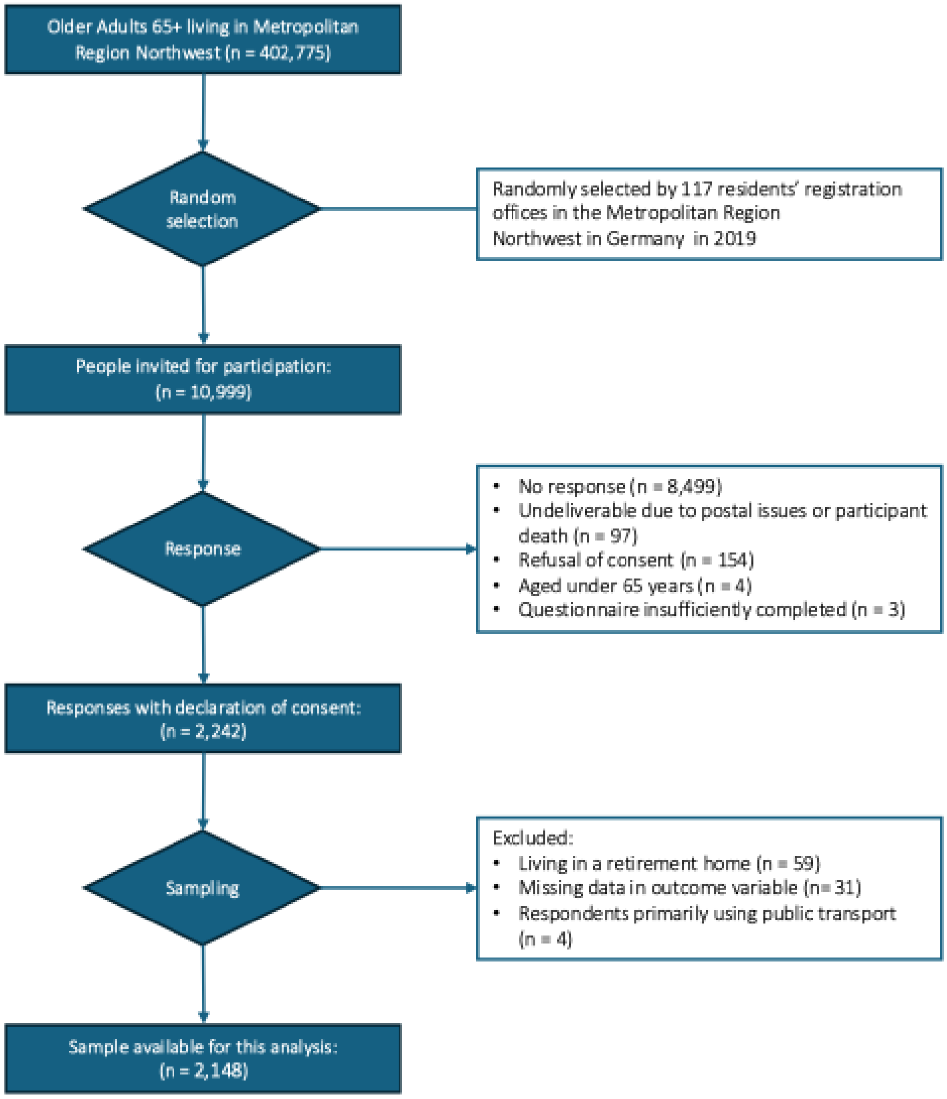

Supplemental Figure 1: Process of selection of the study population. Number of participants living in the Metropolitan Region Northwest were drawn from the Regional Monitoring Metropolregion Northwest, 2015 (based on the 2011 Census).

Supplemental Table 1: Statements on the assessed environmental attributes (based on the NEWS questionnaire)

Walking infrastructure

- ‘There are sidewalks on most of the streets in my neighborhood’
- ‘The sidewalks are well maintained (paved, even, and few potholes)’
- ‘The sidewalks are wide enough’

Cycling infrastructure

- ‘There are cycle paths on most of the streets in my neighborhood’
- ‘The cycle paths are well maintained (paved, even, and few potholes)’
- ‘The cycle paths are wide enough’

Street connectivity

- ‘There are many four-way intersections’
- ‘The distance between intersections is usually short (100 meters or less; the length of a soccer field or less)’
- ‘There are many alternative routes for getting from place to place (I don’t have to go the same way every time)’

Aesthetics

- ‘There are trees along the streets in my neighborhood’
- ‘Trees give shade for the sidewalks in my neighborhood’
- ‘There are many interesting things to look at while walking in my neighborhood’
- ‘My neighborhood is generally free from litter’
- ‘There are many attractive natural sights in my neighborhood (such as (front) gardens, landscaping, views)’
- There are attractive buildings/homes in my neighborhood

Traffic safety

- ‘There is a lot of traffic along the street I live in’
- ‘The speed of traffic on the street where I live is usually fast’
- ‘Most drivers exceed the posted speed limits while driving in my street’
- ‘I like to walk along the street where I live’
- ‘On this street, I feel safe from road accidents’
- ‘Crossing the road is safe for pedestrians’

Crime safety

- ‘On this street, I feel safe from crime’

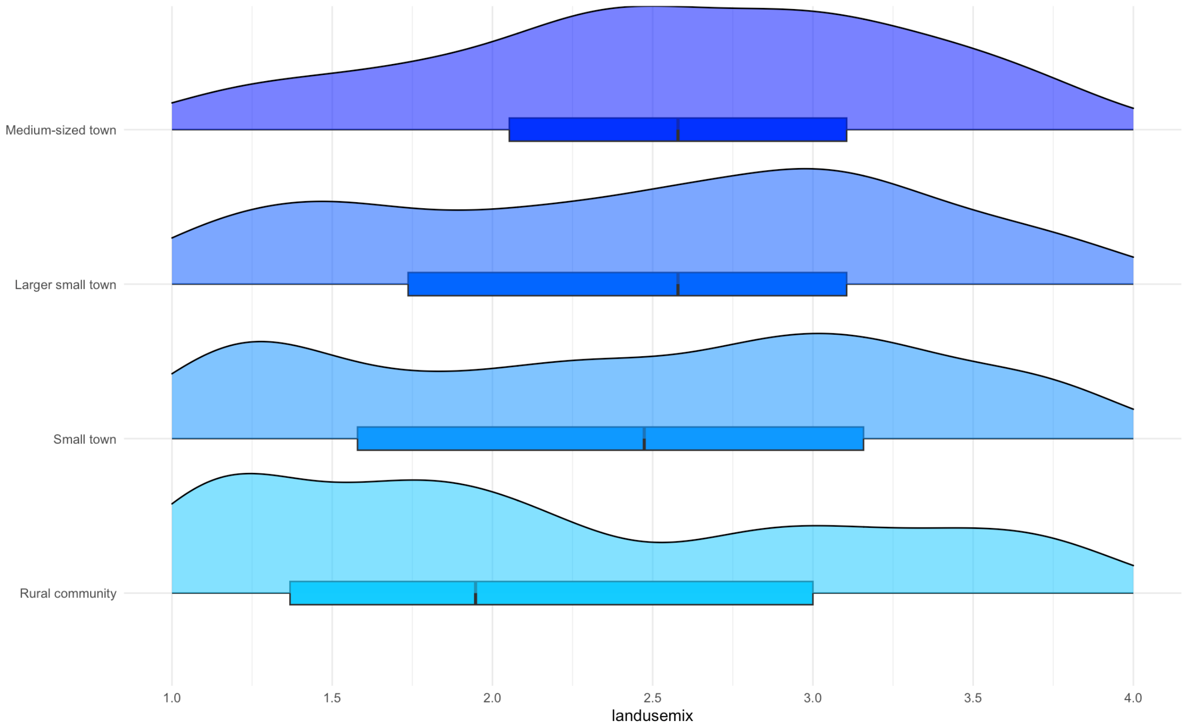

Supplemental Figure 2: Degree of land use stratified by the four categories of municipalities (according to their population size). Mean (sd): Medium-sized town: 2.56 (0.73), Larger small town: 2.47 (0.82), Small town: 2.41 (0.88), Rural community: 2.19 (0.90)

Supplemental Table 2: Description of splitting variables

	total	gender	
		women	men
	2148	976 (47.0)	1099 (53.0)
sociodemographic variables and living situation			
age (years)			
65–69	584 (28.1)	285 (29.2)	299 (27.2)
70–74	531 (25.6)	257 (26.3)	273 (24.8)
75–79	497 (24.0)	230 (23.6)	267 (24.3)
80+	462 (22.3)	204 (20.9)	258 (23.5)
missings	74		
education			
low	165 (7.8)	126 (12.9)	34 (3.1)
middle	1207 (57.0)	622 (63.7)	536 (48.8)
high	750 (35.3)	217 (22.2)	514 (46.8)
missings	26		
equivalized income			
< 60% median	280 (13.4)	130 (13.3)	143 (13.0)
60% - median	688 (33.0)	328 (33.6)	332 (30.2)
>median	1118 (53.6)	485 (49.7)	596 (54.2)
missings	62		
household composition			
living alone	448 (20.9)	267 (27.4)	164 (14.9)
not living alone	1700 (79.1)	709 (72.6)	935 (85.1)
health and mobility restrictions			
self-rated health			
very good/good	1244 (58.5)	560 (57.4)	631 (57.4)
moderate – very poor	881 (41.5)	406 (41.6)	455 (41.4)
missings	23		
mobility restrictions			
yes	812 (38.3)	385 (39.4)	411 (37.4)
no	1307 (61.7)	572 (58.6)	680 (61.9)
missings	29		
neighborhood environment			
landusemix	2.5 (0.8)	2.4 (0.8)	2.6 (0.8)
walking infrastructure	3.3 (0.9)	3.3 (0.9)	3.3 (0.9)
cycling infrastructure	2.3 (0.8)	2.3 (0.8)	2.3 (0.8)
street connectivity	2.7 (0.7)	2.7 (0.7)	2.7 (0.7)
aesthetics	2.8 (0.8)	2.8 (0.8)	2.7 (0.8)
traffic safety	3.2 (0.8)	3.2 (0.8)	3.2 (0.7)
crime safety			
strongly disagree	47 (2.3)	17 (1.8)	26 (2.5)
disagree	84 (4.1)	40 (4.3)	40 (3.8)
agree	678 (32.8)	294 (31.3)	362 (31.3) (34.2)
strongly agree	1261 (69.9)	588 (62.6)	632 (59.6)
missings	78		

N (%), mean (sd) for neighborhood environment

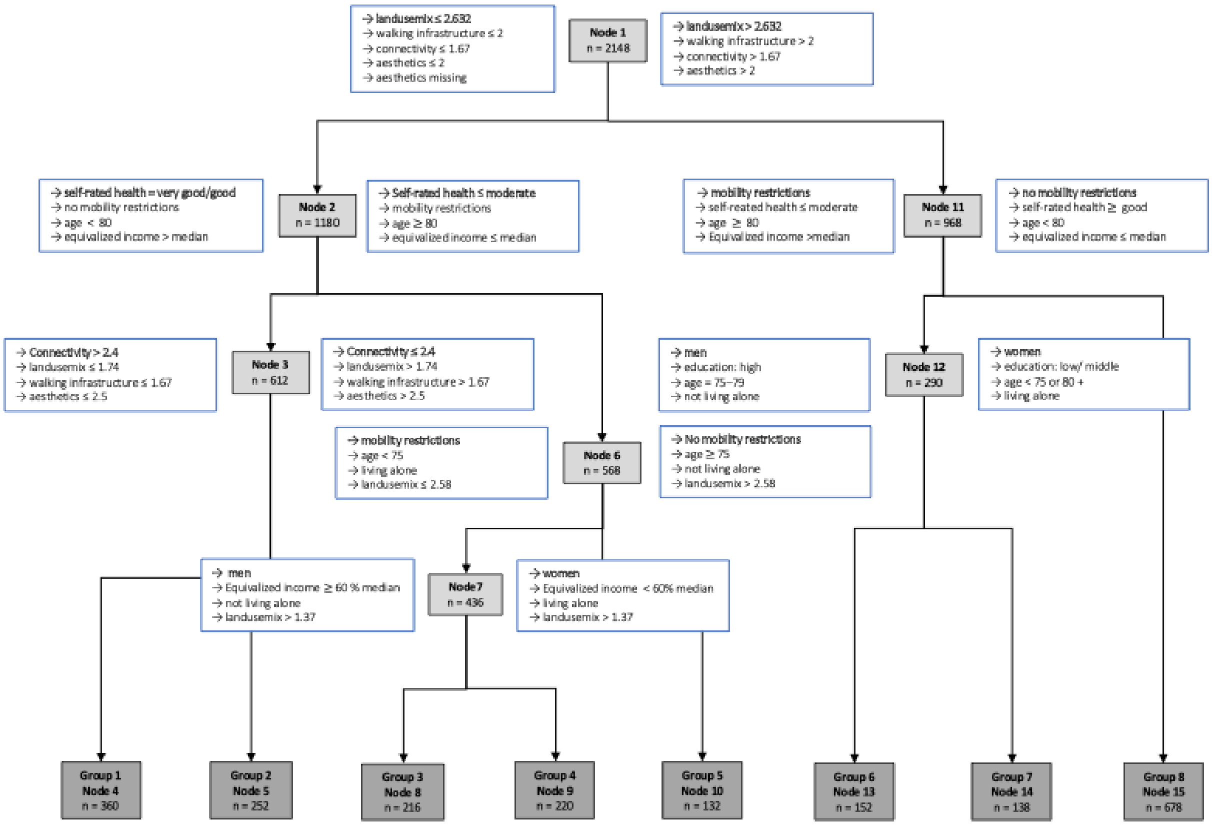

Supplemental Figure 3: Surrogate Splits

Supplemental Table 3: Descriptive analysis per subgroup

	Group 1	Group 2	Group 3	Group 4	Group 5	Group 6	Group 7	Group 8
**node number**	4	5	8	9	10	13	14	15
**mobility type**								
walking	63 (17.5)	61 (24.2)	50 (23.1)	57 (25.9)	38 (28.8)	43 (28.3)	61 (44.2)	179 (26.4)
cycling	65 (18.1)	51 (20.2)	30 (13.9)	14 (6.4)	15 (11.4)	27 (17.8)	26 (18.8)	91 (13.4)
walking + cycling	72 (20.0)	72 (28.6)	28 (13.0)	10 (4.5)	24 (18.2)	41 (27.0)	20 (14.5)	303 (44.7)
car-centric	134 (37.2)	56 (22.2)	73 (33.8)	76 (34.5)	45 (34.1)	31 (20.4)	18 (13.0)	83 (12.2)
not very active	25 (6.9)	11 (4.4)	23 (10.6)	46 (20.9)	9 (6.8)	4 (2.6)	10 (7.2)	19 (2.8)
not active at all	1 (0.3)	1 (0.4)	12 (5.6)	17 (7.7)	1 (0.8)	6 (3.9)	3 (2.2)	3 (0.4)
**drivers license**								
yes	344 (97.7)	238 (95.2)	201 (94.8)	167 (78.8)	122 (93.1)	144 (95.4)	111 (81.6)	638 (96.7)
no	8 (2.3)	12 (4.8)	11 (5.2)	45 (21.2)	9 (6.9)	7 (4.6)	25 (18.4)	22 (3.3)
**access to car**								
yes	348 (98.6)	243 (97.6)	203 (95.8)	199 (93.0)	129 (98.5)	143 (94.7)	122 (90.4)	650 (98.5)
no	5 (1.4)	6 (2.4)	9 (4.2)	15 (7.0)	2 (1.5)	8 (5.3)	13 (9.6)	10 (1.5)
**gender**								
women	178 (50.7)	121 (50.2)	219 (100)	0	53 (41.7)	0	132 (100)	273 (42.3)
men	173 (49.3)	120 (49.8)	0	211 (100)	74 (58.3)	149 (100)	0	372 (57.7)
**age**								
mean (sd)	73.2 (6.3)	73.4 (6.0)	77.3 (7.0)	77.4 (7.6)	73.4 (5.8)	76.7 (6.6)	76.3 (6.9)	72.9 (5.2)
65–69	131 (37.4)	78 (32.4)	36 (17,1)	39 (17.8)	41 (32.3)	26 (17.4)	25 (18.8)	208 (32.2)
70–74	87 (24.9)	68 (28.2)	40 (19,0)	43 (19.6)	38 (29.9)	26 (17.4)	37 (27.8)	192 (29.8)
75–79	71 (20.3)	56 (23.2)	48 (22,9)	50 (22.8)	25 (19.7)	47 (31.5)	25 (18.8)	175 (27.1)
80+	61 (17.4)	39 (16.2)	86 (41.0)	87 (39.7)	23 (18.1)	50 (33.6)	46 (34.6)	70 (10.9)
**education**								
low	26 (7.3)	14 (5,6)	7 (3.3)	51 (23.7)	12 (9.2)	9 (6.0)	21 (15.3)	25 (3.7)
medium	193 (54.2)	140 (56.0)	121 (57.6)	133 (61.9)	79 (60.3)	69 (46.0)	85 (62.0)	387 (57.5)
high	137 (38.5)	96 (38.4)	82 (39.0)	31 (14.4)	40 (30.5)	72 (48.0)	31 (22.6)	261 (38.8)
**equivalized income**								
< 60% median	43 (12.2)	29 (11.8)	50 (24.5)	36 (17.5)	26 (20.2)	22 (14.6)	19 (14.1)	55 (8.3)
60% - median	102 (29.0)	85 (34.6)	75 (36.8)	85 (41.3)	41 (31.8)	56 (37.1)	54 (40.0)	190 (28.7)
>median	207 (58.8)	132 (53.7)	79 (38.7)	85 (41.3)	62 (48.1)	73 (48.1)	62 (45.9)	418 (63.0)

area of residence								
medium-sized town	90 (25.5)	97 (39.1)	60 (28.0)	54 (24.9)	37 (28.7)	47 (30.9)	39 (28.3)	220 (32.8)
larger small town	120 (34.0)	94 (37.9)	76 (35.5)	91 (41.9)	42 (32.6)	58 (38.2)	53 (38.4)	245 (36.6)
small town	105 (29.7)	41 (16.5)	58 (27.1)	54 (24.9)	36 (27.9)	42 (27.6)	36 (26.1)	166 (24.8)
rural community	38 (10.8)	16 (6.5)	20 (9.3)	18 (8.3)	14 (10.9)	5 (3.3)	10 (7.2)	39 (5.8)

N (%)

Supplemental Table 4: Mobility restrictions per group

	Group 1	Group 2	Group 3	Group 4	Group 5	Group 6	Group 7	Group 8
Node number	4	5	8	9	10	13	14	15
**mobility restrictions**	52 (14.6)	39 (15.7)	216 (100)	219 (99.6)		151 (99.3)	135 (97.8)	
**kind of mobility restriction**								
walking impairment	16 (4.4)	22 (8.7)	137 (63.4)	148 (67.3)		90 (59.2)	74 (53.6)	
visual impairment	7 (1.9)	7 (2.8)	30 (13.9)	33 (15.0)		17 (11.2)	17 (12.3)	
other impairments	30 (8.3)	13 (5.2)	105 (48.6)	94 (42.7)		66 (43.4)	64 (46.4)	
**number of mobility restrictions**								
at least 1	51 (14.2)	37 (14.7)	167 (77.3)	167 (75.9)		129 (84.9)	116 (84.1)	
at least 2	1 (0.3)	1 (0.4)	42 (19.4)	48 (21.8)		22 (14.5)	18 (13.0)	
at least 3	0	1 (0.4)	7 (3.2)	4 (1.82)		0	1 (0.7)	

N (%), groups 5 and 8 do not contain any participants with mobility restrictions

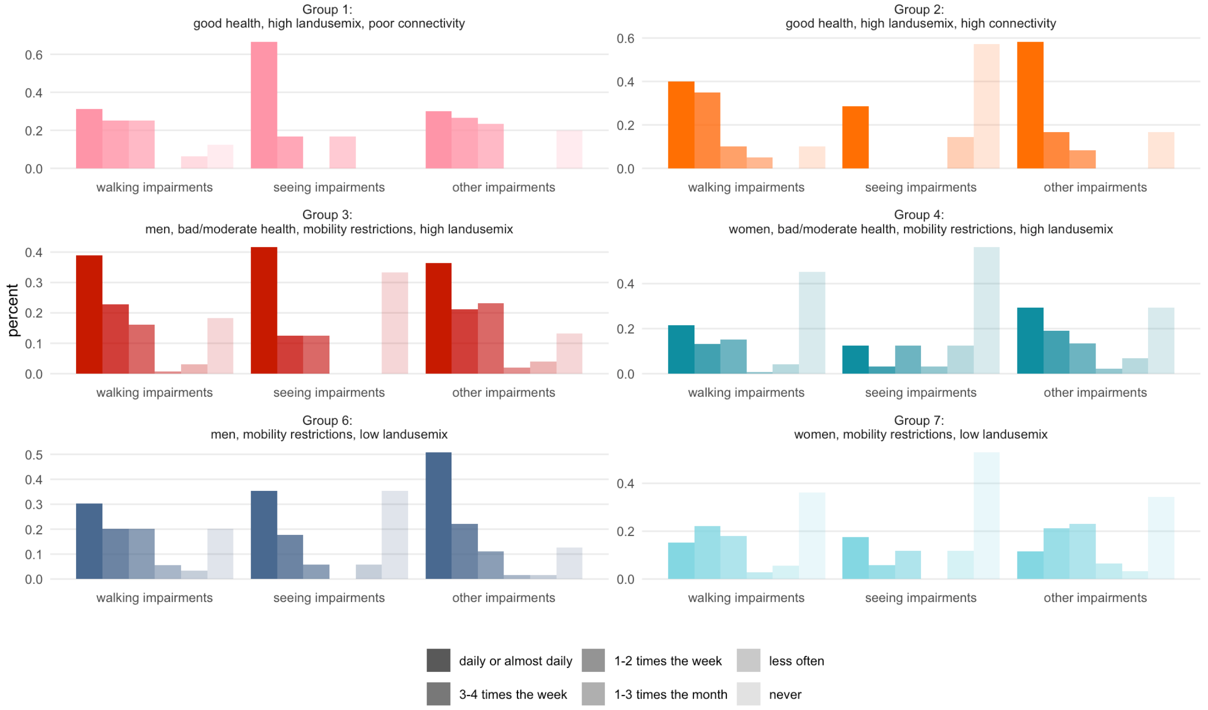

Supplemental Figure 4: Frequency of car use as a driver and mobility restrictions per subgroup, groups 5 and 8 do not contain any participants with mobility restrictions

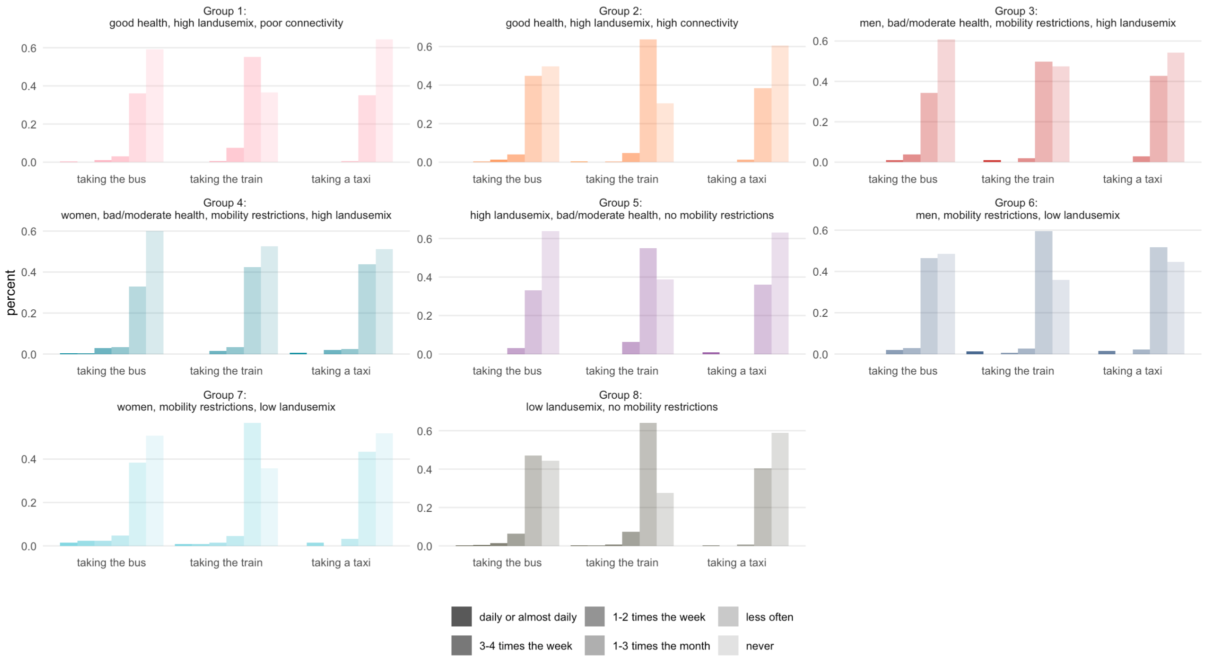

Supplemental Figure 5: Public transport use per subgroup

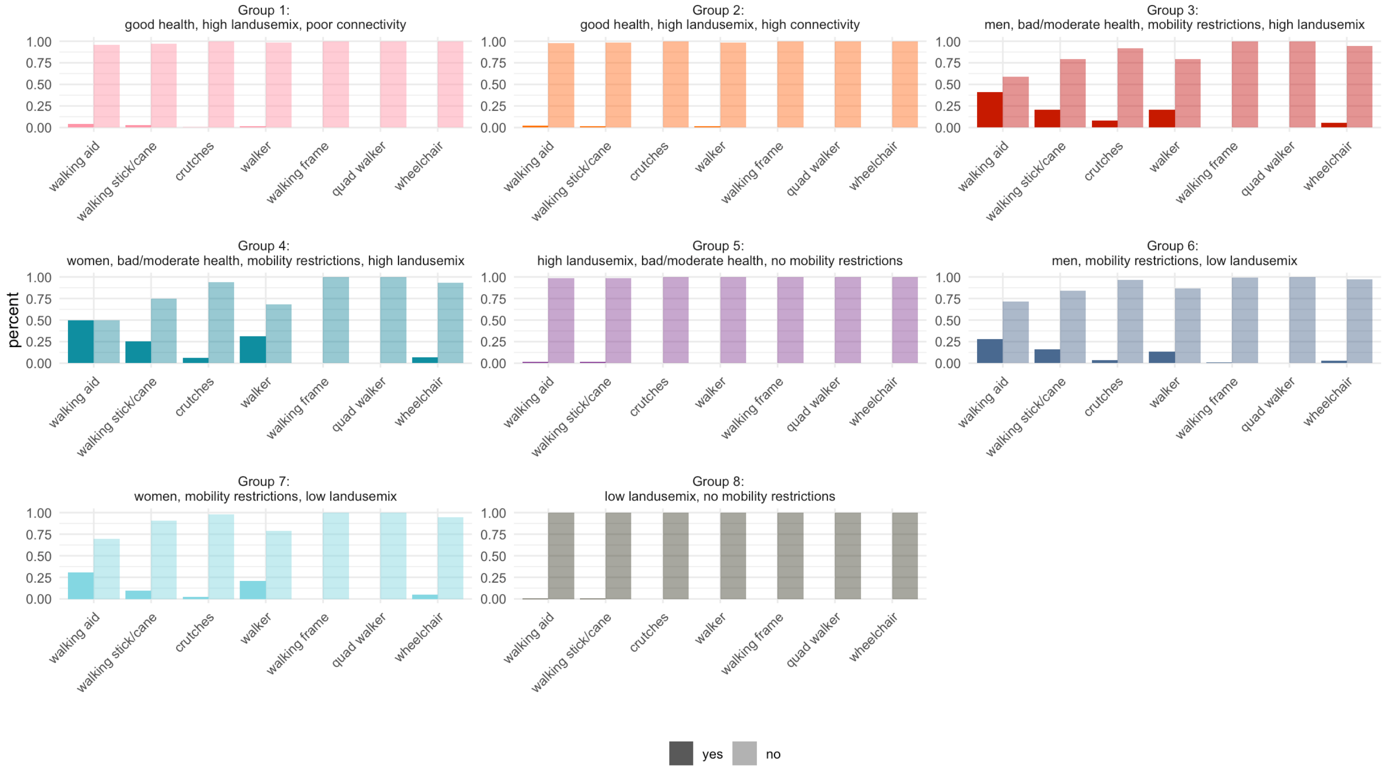

Supplemental Figure 6: Use of walking aids per subgroup. The first column shows walking aid use overall, which is further specified by type in the following columns

Supplemental Table 5: Percentage of study participants rating features of their neighborhood environment as important per subgroup

	Group 1	Group 2	Group 3	Group 4	Group 5	Group 6	Group 7	Group 8
good surface quality	64.2	66.0	63.6	76.8	59.8	73.6	84.4	74.6
seating availability	18.4	23.7	26.8	42.2	25.6	29.3	41.7	22.6
availability of waste bins	25.1	41.0	32.4	41.5	34.4	40.5	51.5	39.5
barrier-free accessibility of paths	45.3	55.7	56.2	70.7	52.8	68.0	81.2	61.9
availability of public restrooms	14.7	20.8	18.5	26.9	19.4	26.9	24.8	24.3
good lighting	61.1	72.0	57.3	69.7	69.5	74.5	86.4	73.9
attractive ground-floor area	7.4	9.8	6.1	14.5	7.3	12.2	13.0	12.7
attractive buildings	13.4	15.2	6.1	18.4	14.5	17.7	20.5	15.6
planting along the paths	43.6	49.8	38.9	44.7	42.9	42.8	53.0	49.9
plenty of room for walking	54.3	64.2	57.0	67.8	50.4	64.1	77.8	59.9
limited motorized traffic volume	38.2	47.1	34.6	44.2	37.6	42.0	52.7	40.1
low traffic speed	52.3	60.6	54.9	57.8	52.3	56.4	65.9	59.1
safety from crime	74.3	83.1	71.4	81.8	74.2	78.5	90.4	84.7
road traffic safety	72.5	83.9	69.7	79.1	78.6	77.1	87.3	84.2
safe crossings	58.0	70.7	50.9	71.8	59.1	64.1	85.3	73.8
separate lanes for pedestrians and bicycles	34.2	44.8	39.0	54.8	41.3	38.4	54.8	43.5
car parking availability	34.3	49.2	39.1	49.0	40.8	46.2	51.9	48.1
bike parking availability	27.5	37.8	25.1	34.7	31.7	36.4	47.4	38.6
navigation aids	42.7	50.0	37.4	50.5	43.5	49.0	59.0	49.2
drinking water dispenser	5.4	4.5	5.7	11.0	5.7	5.6	10.4	4.2
public spaces	22.4	34.1	25.6	41.5	25.2	31.3	39.0	32.0

Supplemental Table 6: Percentage of study participants rating features of their neighborhood environment as important when choosing their mode of transport per subgroup

	Group 1	Group 2	Group 3	Group 4	Group 5	Group 6	Group 7	Group 8
predictable time	57.1	63.5	55.3	69.6	56.6	62.4	70.5	57.3
short time	43.1	45.2	49.5	67.0	47.7	60.0	66.2	47.0
low costs	42.1	47.6	47.1	51.2	46.5	53.1	57.0	47.7
high comfort	21.0	18.5	20.9	20.1	18.8	19.6	25.8	18.7
safety from crime	65.5	72.0	60.1	70.9	68.2	73.3	80.7	70.6
safety from traffic	69.5	76.4	64.7	73.3	68.8	74.0	88.1	73.7
safety from falling	65.4	72.6	70.2	81.2	66.4	78.0	88.1	68.3
low air pollution	52.1	63.1	53.1	52.2	46.5	65.3	68.1	58.4
low noise	42.9	56.4	48.1	44.4	41.1	56.7	63.4	51.2
privacy	25.4	21.8	18.8	29.3	21.9	22.3	25.9	18.0
promotion of health	57.9	70.2	61.4	68.1	61.2	69.3	71.4	63.4
environmentally friendly	53.1	61.0	50.7	54.6	51.2	58.9	67.9	58.4
flexible departure times	49.3	54.9	45.6	56.9	53.1	48.6	54.5	49.5
protection from weather	47.9	51.6	47.6	67.1	53.8	48.0	59.3	48.5

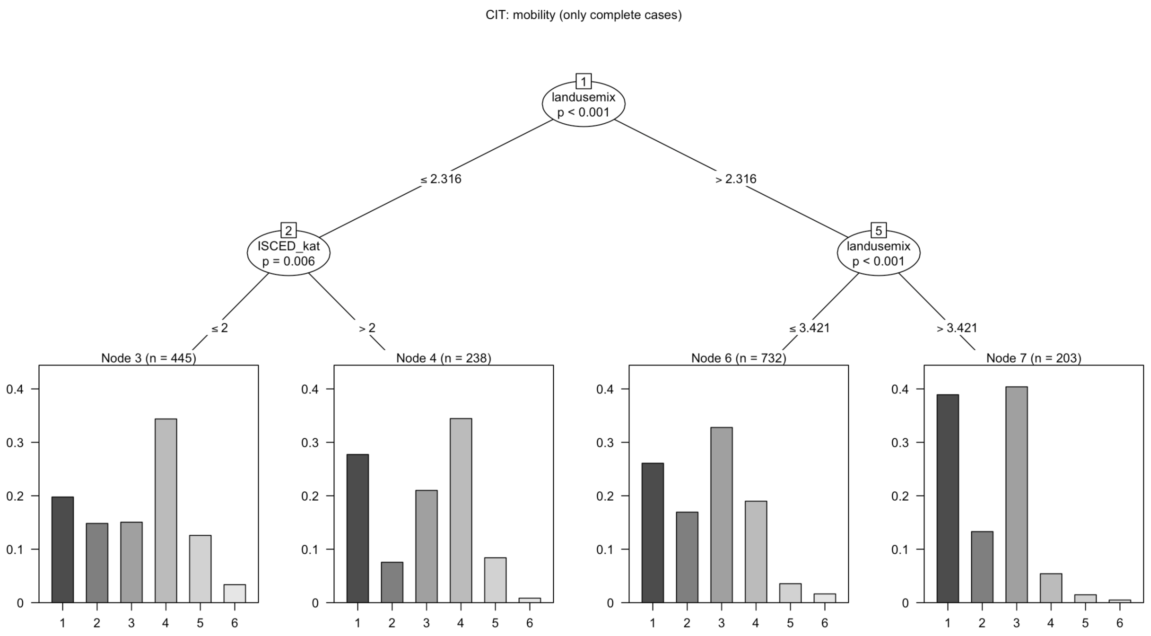

Supplemental Figure 7: Sensitivity analysis: Decision Tree with complete cases (n = 1.618)

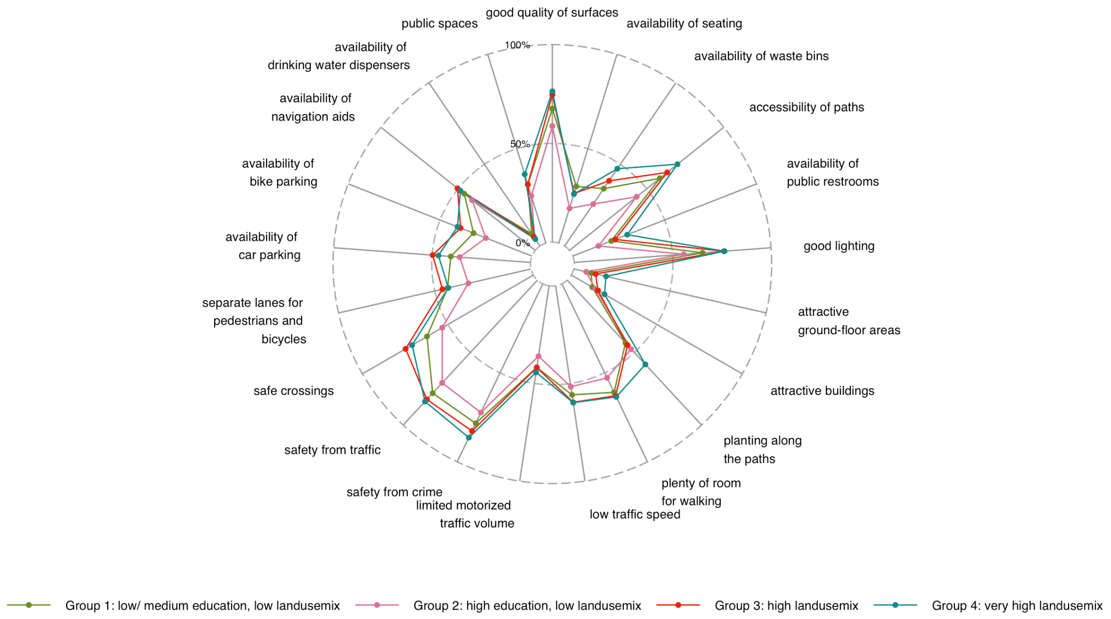

Supplemental Figure 8: Sensitivity analysis: complete cases (n = 1.618), Proportion of participants per subgroup who rated features of their structural and spatial neighborhood environment as important

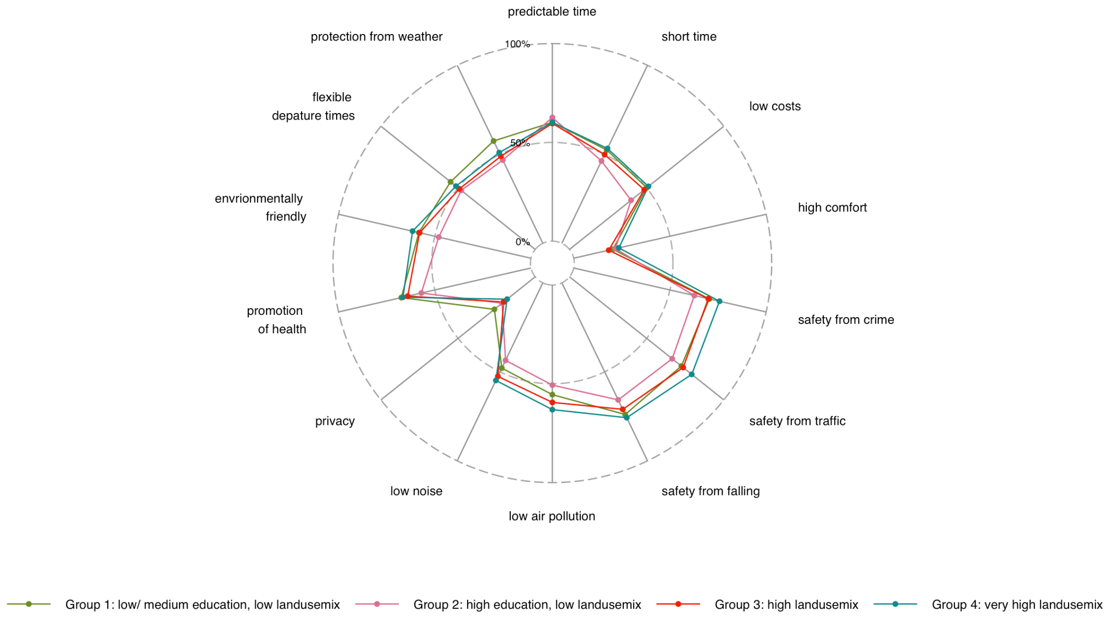

Supplemental Figure 9: Sensitivity analysis: complete cases (n = 1.618), Proportion of participants per subgroup who rated various features of the neighborhood environment as important when choosing their mode of transport

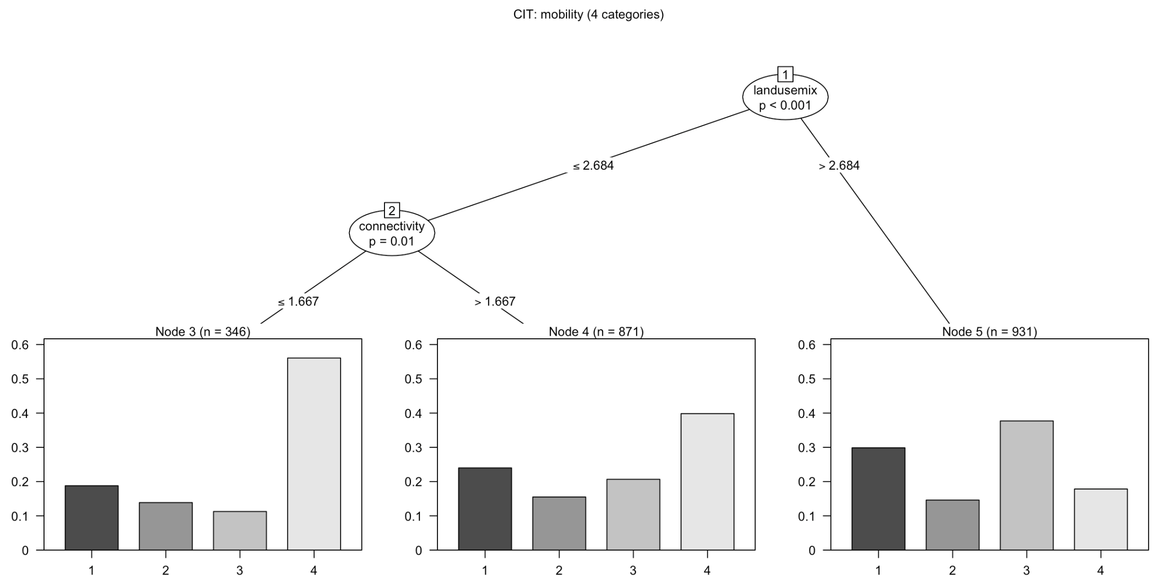

Supplemental Figure 10: Sensitivity analysis: Decision Tree, mode of transport categorized into four categories

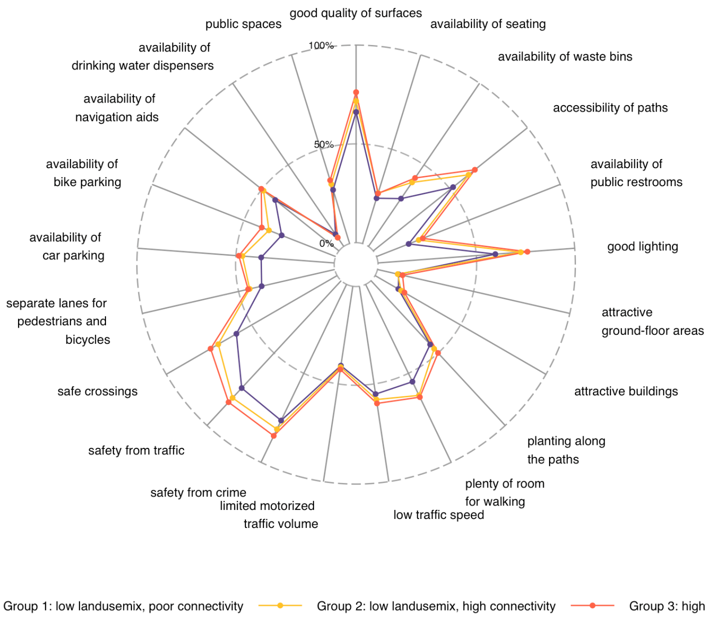

Supplemental Figure 11: Sensitivity analysis: mode of transport categorized into four categories, proportion of participants per subgroup who rated features of their structural and spatial neighborhood environment as important

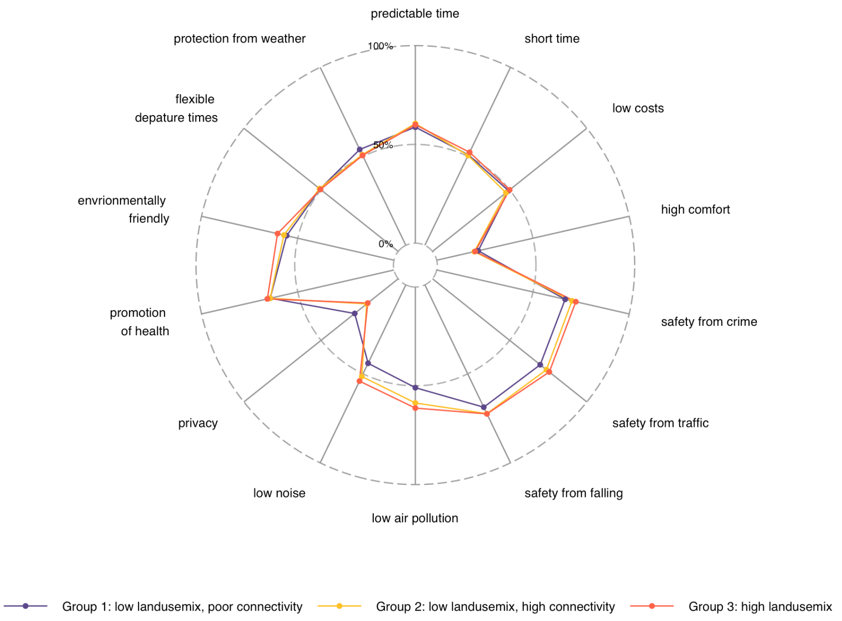

Supplemental Figure 12: Sensitivity analysis: mode of transport categorized into four categories, proportion of participants per subgroup who rated various features of the neighborhood environment as important when choosing their mode of transport

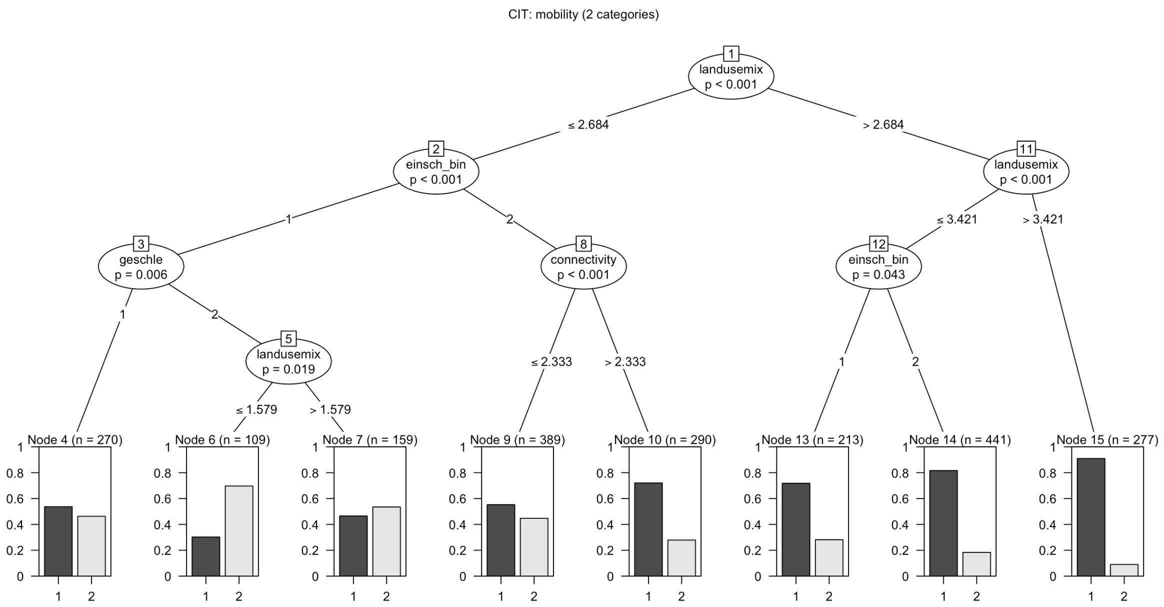

Supplemental Figure 13: Sensitivity analysis: Decision tree, mode of transport categorized into two categories

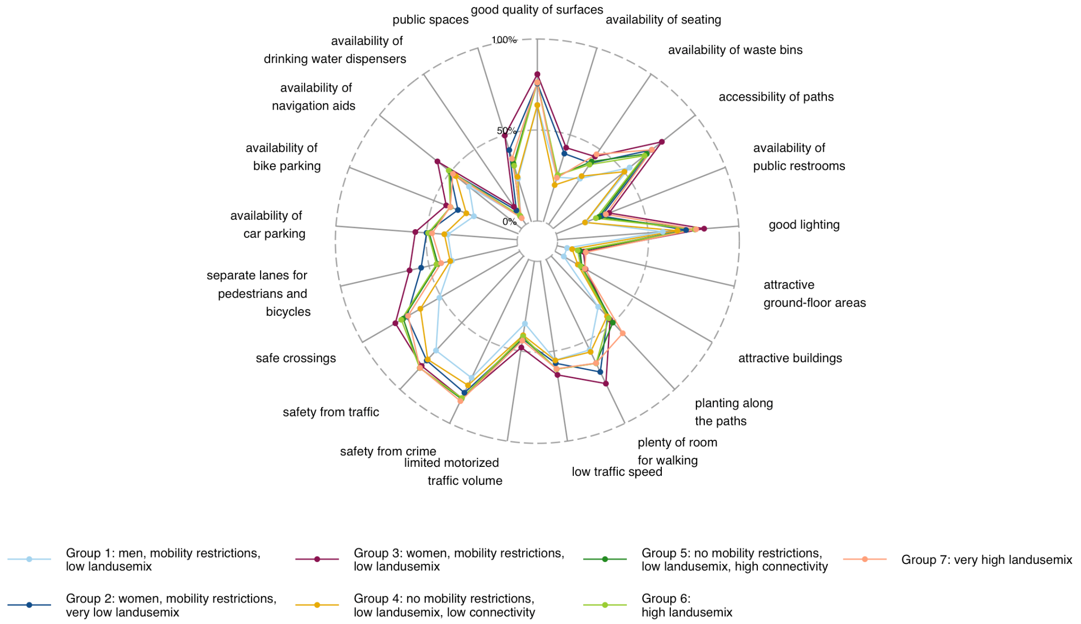

Supplemental Figure 14: Sensitivity analysis: mode of transport categorized into two categories, proportion of participants per subgroup who rated features of their structural and spatial neighborhood environment as important

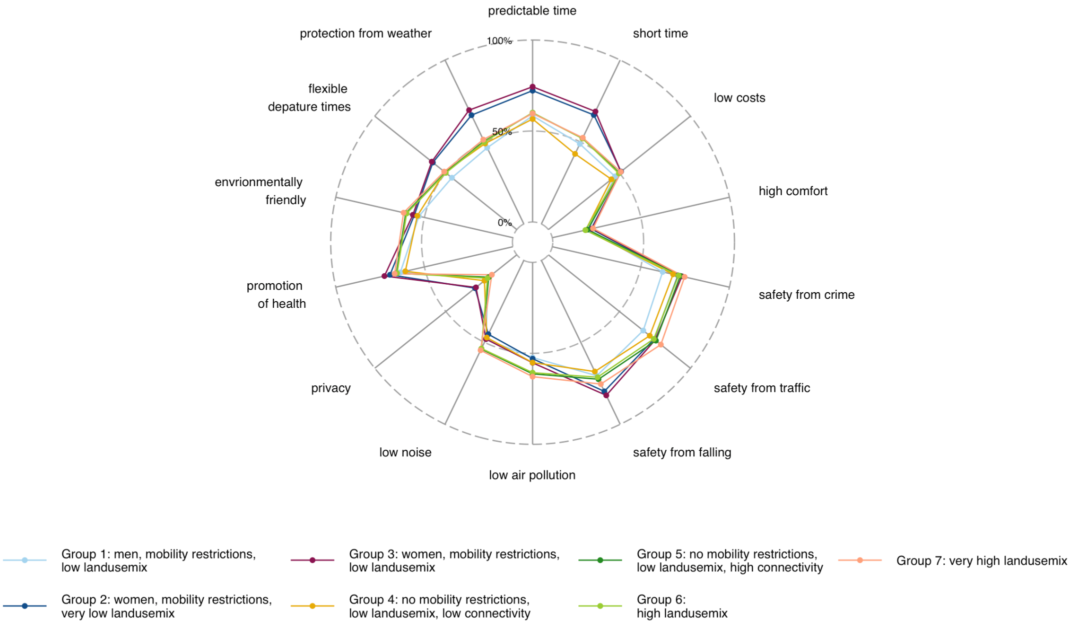

Supplemental Figure 15: Sensitivity analysis: mode of transport categorized into two categories, proportion of participants per subgroup who rated various features of the neighborhood environment as important when choosing their mode of transport
